# Network Threat Detection Using Machine/Deep Learning in SDN-Based Platforms: A Comprehensive Analysis of State-of-the-Art Solutions, Discussion, Challenges, and Future Research Direction

**DOI:** 10.3390/s22207896

**Published:** 2022-10-17

**Authors:** Naveed Ahmed, Asri bin Ngadi, Johan Mohamad Sharif, Saddam Hussain, Mueen Uddin, Muhammad Siraj Rathore, Jawaid Iqbal, Maha Abdelhaq, Raed Alsaqour, Syed Sajid Ullah, Fatima Tul Zuhra

**Affiliations:** 1 School of Computing, Faculty of Engineering, Universiti Teknologi Malaysia, Johor Bahru 81310, Johor, Malaysia; 2 School of Digital Science, University Brunei Darussalam, Jalan Tungku Link, Gadong BE1410, Brunei; 3 College of Computing and Information Technology, University of Doha For Science and Technology, Doha 24449, Qatar; 4 Department of Computer Science, Capital University of Science and Technology, Islamabad 44000, Pakistan; 5 Department of Information Technology, College of Computer and Information Sciences, Princess Nourah bint Abdulrahman University, P.O. Box 84428, Riyadh 11671, Saudi Arabia; 6 Department of Information Technology, College of Computing and Informatics, Saudi Electronic University, Riyadh 93499, Saudi Arabia; 7 Department of Information and Communication Technology, University of Agder (UiA), N-4898 Grimstad, Norway

**Keywords:** software defined network, intrusion detection systems, machine learning, deep learning, security attacks

## Abstract

A revolution in network technology has been ushered in by software defined networking (SDN), which makes it possible to control the network from a central location and provides an overview of the network’s security. Despite this, SDN has a single point of failure that increases the risk of potential threats. Network intrusion detection systems (NIDS) prevent intrusions into a network and preserve the network’s integrity, availability, and confidentiality. Much work has been done on NIDS but there are still improvements needed in reducing false alarms and increasing threat detection accuracy. Recently advanced approaches such as deep learning (DL) and machine learning (ML) have been implemented in SDN-based NIDS to overcome the security issues within a network. In the first part of this survey paper, we offer an introduction to the NIDS theory, as well as recent research that has been conducted on the topic. After that, we conduct a thorough analysis of the most recent ML- and DL-based NIDS approaches to ensure reliable identification of potential security risks. Finally, we focus on the opportunities and difficulties that lie ahead for future research on SDN-based ML and DL for NIDS.

## 1. Introduction

Over the last two decades, network technologies have tremendously improved; at the same time, network security threats have also increased. Web-based security attacks, denial-of-service (DoS), and malicious insiders are a few examples that cause the devastating cybercrimes. With such malicious activities, critical disruptions can occur within a network. To ensure network security, antivirus software, firewalls, and network intrusion detection systems (NIDS) can be deployed. Among these, NIDS is broadly used for detecting intruders within a network by continuously monitoring the network traffic for any suspicious and malicious behavior. NIDS is useful to detect different kinds of network threats, including distributed denial-of-service (DDoS) attacks, worms, and viruses. Reliability, accuracy, and detection speed are the success factors of NIDS. Enormous research work has been done on NIDS, but it still requires improvements in reducing false alarm and increasing detection accuracy. To reduce false alarm rate [1] and increase threat detection accuracy [2], different approaches of machine learning (ML) have been used in NIDS. The advanced type of ML that is deep learning (DL) is also used in developing a more advanced field of NIDS.

Software defined networking (SDN) has revolutionized network technology in recent years. In contrast to a traditional network, SDN decouples the control plane and data plane of a network switch. In SDN, the control plane is moved to a remote controller (server), which can add packet forwarding rules in network switches according to a given program. This central control of a network offers more programmability and visibility compared with a traditional network. In addition, it is also attractive from a network security perspective, as having central control can offer better network monitoring [3].

In SDN, innovative network applications can be developed to monitor and control the network. In this regard, NIDS is extended for SDN-based architecture. To enhance network security and traffic monitoring, different approaches of ML/DL can be implemented in the controller of SDN. From the past few years, the invention of graphics processor units (GPUs) has increased the popularity of ML/DL approaches in network security. Both ML and DL techniques are very efficient at predicting any malicious or suspicious behavior from network traffic, as they can extract and learn new features from network traffic. ML-based NIDS heavily depend upon the learned features from network traffic, whereas DL-based NIDS automatically learn from the raw data of complex features and do not rely on learned features [4].

Many researchers have worked on ML- and DL-based NIDS in order to improve its performance in detecting network intruders. However, in larger networks, security threats are also increased due to increased network traffic, which affects the efficiency of NIDS in detecting malicious activities. Very few studies have been conducted on developing SDN-based NIDS systems through DL approaches so that there is enough room to deploy these techniques for improving detection efficiency of intrusions within a network.

The basic purpose of this review paper is to comprehensively review the current advancements and trends in ML/DL-based NIDS systems and, more significantly, to provide an overview of the work on SDN-based NIDS systems using ML/DL approaches. This paper covers the area of knowledge for people with basic to moderate knowledge related to ML and DL for network threat detection in SDN. The motivation is to provide an overall picture of the existing research outcomes in this area. In addition, we aim to identify future research directions that may be useful for new researchers who are interested in this field of study.

We analyzed the scientific research carried out on network threat detection (NTD) in SDN, based on ML and DL mechanisms. We covered the main and sub-parts of the NTD paradigm to efficiently cover threat issues and their protection using ML and DL approaches to avoid adversary attacks and protect sensitive information during storage and transmission on a public network. There were various review papers covering different aspects of this domain, leveraging ML/DL approaches [5,6,7,8,9]. Much research work has been done on NIDS using ML and DL approaches, but there are few studies on SDN-based NIDS and very few on DL-based NIDS systems in SDN.

We believe our study is different from existing studies for the following reasons (and thus, are the main contributions of our work):

- First, we conducted a comprehensive review on ML/DL-based network intrusion detection systems;
- Second, we reviewed each study on SDN-based NID systems using ML and DL algorithms;
- We also explored recent advancements and trends in ML/DL approaches for NIDS, followed by the NIDS system leveraging SDN using ML/DL approaches, and research issues in NID systems using ML/DL approaches.

This is a review paper and the scope of our work was to include all studies that aimed to identify and/or fix network threats in SDN, using either ML or DL algorithms for threat detection. Table 1 shows a comparison of the current study with similar previously published articles.

The remaining sections of this review paper are as follows: Section 2 provides background knowledge for SDN and NIDS. Section 3 describes ML/DL-based NIDS and evaluates related studies. In Section 4, we review the SDN-based NID system using ML/DL approaches and evaluate related studies. Lastly, we present some open research challenges with ongoing discussion, followed by conclusion and future directions.

## 2. Background

This section includes basic background knowledge for SDN and IDS.

### 2.1. General Architecture of SDN

SDNs configure the whole network through programming, with a central location, as per organizational business needs. SDN is a network emerging paradigm adopted by telecommunication industries including Cisco Systems and Google. It decouples the control plane (i.e., intelligence of a network) from the data plane. A SDN-enabled network device (such as a router or network switch) performs forwarding only (i.e., data plane), whereas a remote controller implements the control plane. As a result, the controller controls the entire network and maintains a global view of the whole network. The separation of control and data planes in SDN can enhance the visibility, adaptability, and other local security operations of the network.

Learning and teaching have been profoundly impacted by the development of technology and proliferation of the Internet. E-learning has emerged from these developments and is generally understood as “the use of computer network technology, typically through an intranet or over the internet, to provide information and instruction to people.” However, e-learning faces several obstacles, such as the wide variety of learning styles and complications that may arise from cultural differences [14].

A SDN-enabled network device maintains a flow table that is consulted to perform a forwarding decision for an incoming packet. An incoming packet is matched against a flow table entry. This matching can be performed on the different header values (such as IP address and port number) of an incoming packet. For a matching flow, an action is listed in the flow table. For instance, a particular packet could either be dropped, forwarded on a particular output port, or forwarded to the controller.

Flow table entries are populated by the external controller. Within the application or module running on the controller, the forwarding rules are defined. To modify the data plane with the help of an external application, an application programming interface (API) is offered by SDN. In SDN, the capabilities of controlling the network have increased compared with traditional networks. The reason for this is that SDN implements a flow-based structure. As SDN is software-based, it is easy to modify policies in SDN that are less prone to errors. As it is programming-based, complex functions in the network can be developed in simpler ways [7].

**Figure 1:**
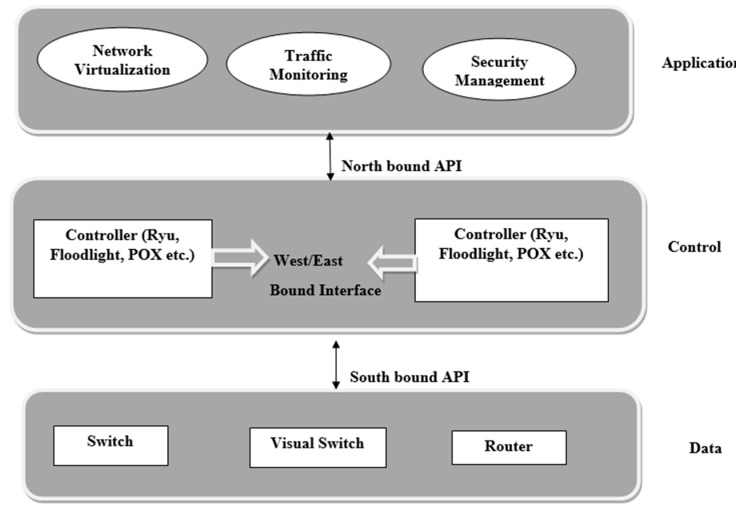
General architecture of SDN.

The general architecture of the SDN is shown in Figure 1. It shows the three planes of SDN, named the application plane, control plane, and data plane, which are discussed in the following sections, respectively.

#### 2.1.1. Data Plane

Another name for the data plane is the infrastructure layer. Normally, there are various network devices in the data plane, including virtual switches and physical switches that are interconnected with each other through wireless or wired media. The data plane is responsible for sending network traffic on to predefined destination by the control plane, which is also called the forwarding plane. Virtual switches are based on software that can be operated through Linux. Examples of virtual switches implementations are Pantou [15], Indigo, and Open vSwitch [16]. Physical switches are based on hardware. Physical switches can be of two types; one type of physical switch can be implemented on networking hardware, whereas the other type is implemented on open network hardware (e.g., NetFPGA [17]). Two open network hardware-based physical switches are ServerSwitch [18] and SwitchBlade [19]. These data plane switches work according to received policies from the control plane, and as a result, they can modify, drop, or forward a packet.

#### 2.1.2. Control Plane

The control layer of the SDN, also named the central layer, is considered the SDN brain, and includes controllers that are responsible for the programming and monitoring of the network and terminating and creating flows. The control layer is also accountable for routing. For example, it identifies the path in which data has to be forwarded using a routing algorithm. On one hand, the control plane extracts information from the data plane and transmits it to application plane; on the other hand, the control plane translates the application plane requirements into policies, and sends these policies to network switches (forwarding elements, FEs). Moreover, the control plane includes features such as providing state information notifications, device configuration, network topology storage, and shortest path routing etc. Beacon, OpenDayLight [20], Ryu, Flood-light [21], POX, and NOX [22] are different kinds of controllers. The control plane can interact within and outside the plane, with the help of three communication interfaces: the westbound/eastbound interface, southbound interface, and northbound interface [23].

#### 2.1.3. Southbound Interface

The southbound interface is responsible for the interaction between the control plane and data plane. Through the southbound interface, a controller communicates with a switch, for instance, to add a new entry in flow table. Apart from forwarding operations, other important information (such as statistics reports and event notifications) are also exchanged through the southbound interface.

#### 2.1.4. The Northbound Interface

The northbound interface is an API communication interface connecting the control layer and application layer. Through the northbound interface, the control plane translates the application plane requirements into policies, and sends these policies to FEs of the data plane [24].

#### 2.1.5. Westbound/Eastbound Interfaces

The westbound/eastbound interfaces are used in SDN containing the multi-controller. When SDN is deployed in large networks, one controller may be unable to process the large amount of network traffic or data flows, so the larger networks are divided into smaller domains with separate controllers dedicated to each domain. Thus, communication between these separate controllers is necessary so that the global network view can be presented to the application plane; this communication is where westbound/eastbound interfaces are used. Table 2 describes threats that occur in SDN planes.

### 2.2. Network Intrusion Detection System

Threats are detected by monitoring packets using IDS. Malicious and abnormal activities are detected by IDS from both the inside and outside [6]. Highly rough distribution of data and vast volumes of network traffic are some problems related to the IDS.

**Figure 2:**
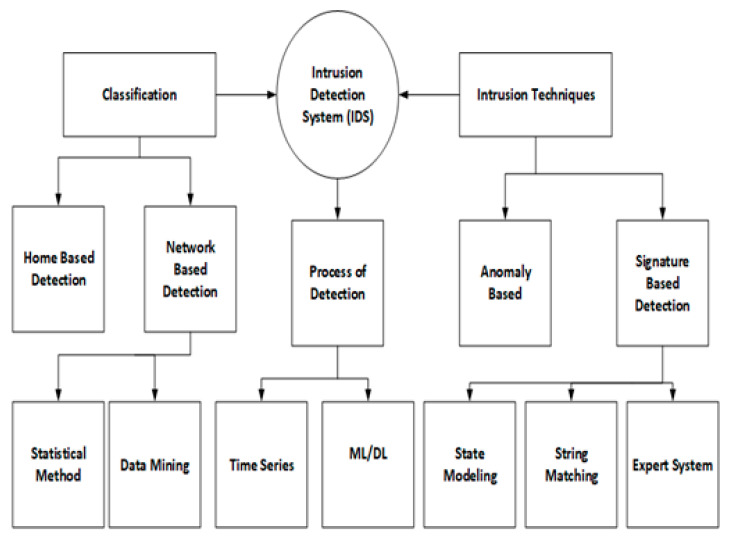
Overview of IDS.

Networks or computers are some of the information sources that are monitored by the IDS, as its main function is to report illegal activities or access. Data from various network sources and systems are collected and analyzed by IDS for all possible threats and attacks. A summary of IDS deployment environments and implemented techniques of detection is shown in Figure 2. As Figure 2 describes, various techniques and methods can be used to implement intrusion detection systems, which are further divided into following groups: ML-based methods, data mining methods, and statistical techniques [8]. IDS has a wide array of implementations, including systems from tiered monitoring systems to antivirus software by which traffic of a complete network is followed. It can be categorized into the following classes:

- Incoming network traffic analyzed by the system known as NIDS.
- Important files of the operating system are monitored by the system and defined as “Host-based intrusion detection systems (HIDS)”.
- The aforementioned classifications of IDS are further classified. Signature and anomaly detection are the basis of commonly used variants [25].

#### 2.2.1. Signature-Based Detection

Possible threats are detected by signature-based IDS by considering some special patterns, such as some known intrusion sequences that are malicious and used by Trojan or some byte sequences used in the traffic of network. This terminology originated from antivirus software that referred to these detected patterns as signatures. Known attacks are easily detected by signature-based IDS, but detection of new attacks for which there is no recognizable pattern is not possible [26].

#### 2.2.2. Anomaly-Based Detection

This is a new technique that was designed for the adaption and detection of unknown attacks mainly caused by explosion of malware. ML is used in this detection method to model a trustworthy activity, and then the new behavior of the newly developed model is compared with the true model. Although unknown attacks are detected by this approach, there is also a risk for false positives, i.e., the classification of unknown authentic activities can be reported as malicious [27]. In [28], an algorithm was designed for an anomaly-based system named the AdaBoost algorithm. In this algorithm, two feature selection approaches, i.e., principal component analysis (PCA) and ensemble feature selection (EFS), were utilized for selecting features from a novel set of data, CICIDS 2018. Experimental results showed that integration of EFS with AdaBoost gave better results compared with PCA with AdaBoost. An analysis related to passing traffic is performed by IDS located at a premeditated point in the network to monitor traffic from network devices, and then the traffic is matched on subnets to a library of all known threats. Once the identification of an attack is made, it senses any abnormal behavior and the administrator receives an alert [28]. Table 2 shows threats on various SDN plans.

There are various other studies as well on IDS. For instance, [29] provides a comprehensive review on IDS, another study [30] suggests a network-based IDS. Similarly, in [31] authors argue how a high-performance IDS can be designed whereas the implementation of IDS using genetic algorithm is discussed in [32].

**Table 2:** Threats on SDN.

Target Plane	Threat	Reason
Control plane	Controller hijacking	Due to malicious application vulnerability leverage in NBI
Application plane	Threats from applications	Lack of authorization and authentication
Control plane	Spoofing	Due to absence of switch and TLS authentication consistency or compromised verification checks in flow rules.
Control plane	MITM attack between controller and switches	Without TLS security, the communication channel is not secured
Data plane	Fingerprinting SDN networks	Difference in time to process packets between SDN and traditional network
Control plane	Denial of service attack to saturate flow table	Centralized controllers
Data plane	Information disclosure	Flow tables limitation
Data plane	Tampering attack using fraud flow rules	Difference in time to process packets, which reveals information about the content of flow
Data plane	ICMP attacks, DoS attacks, sequence prediction attack, reset attack, and SYN attacks	Inheritance of TCP level attacks from traditional networks
Data plane	Cache poisoning attack against the controller state and flow table	Inserting forged packets
Data plane	Freeloading	Spoofing IP/MAC address to one of the hosts of an already established communication link.

## 3. Machine Learning and Deep Learning in NIDS

**Figure 3:**
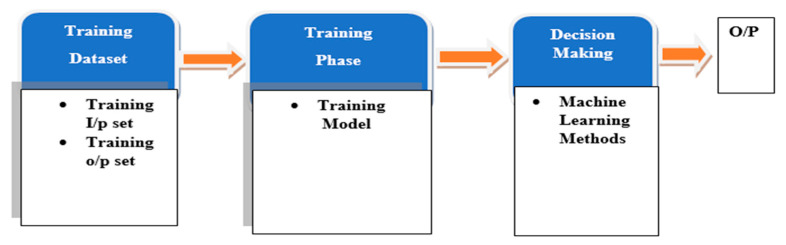
Processing procedure of the ML method.

In the areas of artificial intelligence (AI), there are numerous powerful ML techniques evolved and commonly used in data mining, and useful structural patterns and models from the training set are learnt by the system with the help of ML [33]. There are mostly two phases included in the ML technique: (1) Training phase and (2) Decision-making phase (Figure 3). In the training stage of the ML technique, training data is used by the applied methods of ML to learn the system model. In the second stage, estimated output can be obtained by the system with the help of a trained model for every new input [34].

**Figure 4:**
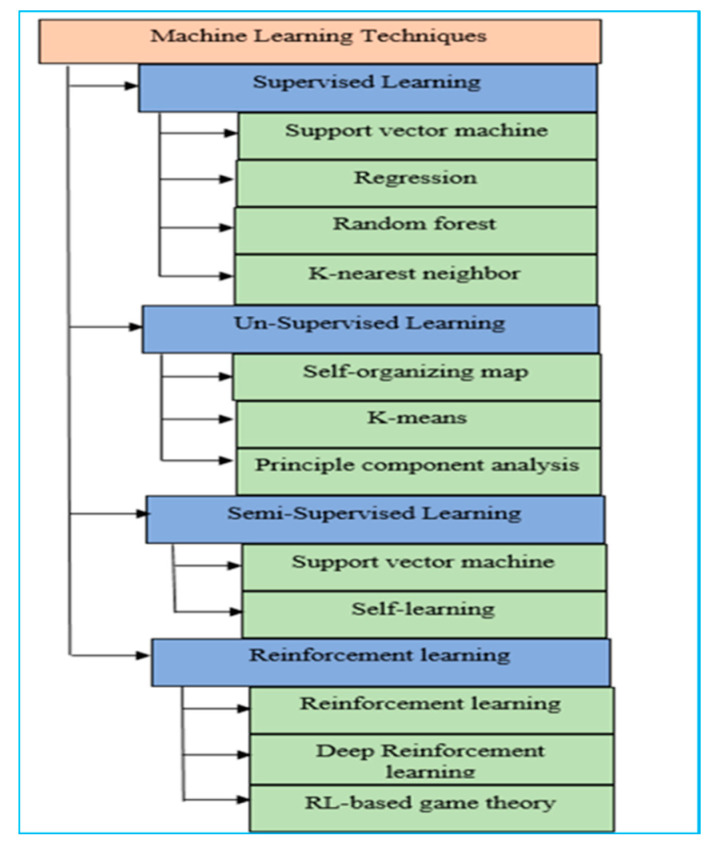
Commonly used approaches of machine learning.

There are basically four types of ML algorithms: supervised learning, reinforcement learning, unsupervised learning, and semi-supervised learning, as given in Figure 4. The commonly used techniques of ML [35] are described in the following subsections. Figure 3 shows the processing procedure of ML methods.

### 3.1. Supervised Learning

To anticipate unknown cases, representations are learnt by the algorithms from labelled input data in supervised learning. Examples of this learning include support vector machine (SVM) and random forest methods, where SVM is used for classification-related problems and problems related to classification and regression are dealt with random forest [36]. In NIDS research, the most commonly used algorithm is SVM, as it is practical in computation and powerful classification abilities. In the case of high dimensional data, SVM algorithms are mostly suitable, but it is very difficult to select an accurate kernel function. Memory and processing units of computation are demanded by SVM [37], so it is resource hungry. In case of uneven data, an effective supervised ML approach involves a random forest algorithm, but it can suffer from the problem of overfitting [38].

#### 3.1.1. Random Forest

Random decision forest is another name of the random forest method, and tasks related to classification and regression are dealt with this method [39]. Different decision trees are included in the random forest model. Problems of overfitting from the decision-tree method can be mitigated with the construction of decision trees by randomly selecting from the feature support subset; in this way. the accuracy of the model can also be improved. The following steps are included in the random forest method for the classification of a new data sample: (1) Put the sample of data to each tree in the forest. (2) Classification results given by each tree is known as the vote of the tree. (3) The sample of data will be part of the class that receives the most votes.

#### 3.1.2. Support Vector Machine

Another important technique of supervised learning is SVM, usually used in tasks related to classification and recognition of patterns. Vapnik and others [40] invented SVM; mapping of the i/p vector into high-dimensional feature support is the main idea behind the SVM. Various kernel functions are applied to achieve mapping, such as radial, polynomial, and linear-based functions. In SVM, selection of kernel function is critical, as it affects the accuracy of classification. The training dataset is the base for the selection of kernel function. If training data is linearly separable, a linear kernel function will be more accurate. Kernel functions for example, Radial based function and polynomial is commonly used in the case when training data is not linearly separable. Generally, accuracy of RBF function is much higher as compared to polynomial and linear kernel functions [41,42]. Further detailed discussion related to SVM classifier is given in [43,44].

#### 3.1.3. k-Nearest Neighbor

**Figure 5:**
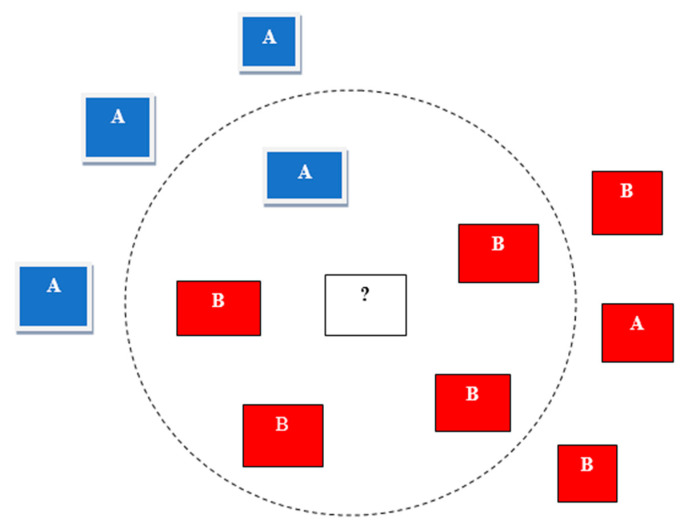
Example of k-NN algorithm (k = 5) [49]. (A and B are the elements of selected sample from a given population to form clusters by using KNN algorithm).

In this supervised learning technique, a sample of data is classified in terms of k-nearest neighbors (k-NN) of the unclassified sample. The classification is performed on the basis of number k-NN; for example, if a sample has more related k-NN, the sample will be classified in that class. A simple and easy example of k-NN is shown in Figure 5, where the working principle of k-NN is also explained. When the value of k is one, it will be the nearest neighbor algorithm. There is a reduced effect of noise on the classification with a higher value of k. In k-NN algorithms, the main factor is distance, so distance can be defined by various functions between the sample of data that is not labelled and its neighbors, including Euclidean, Chebyshev, Euclidean squared, and City-Block functions. A detailed discussion of k-NN can be found in [45].

### 3.2. Unsupervised Learning

Representations are learnt by the algorithms from unlabeled input data in unsupervised learning. The purpose of an unsupervised learning algorithm is to anticipate unknown cases through the distribution or modelling of fundamental structures in the data [46]. PCA and self-organizing maps (SOM) are the main examples of unsupervised learning. PCA is used in techniques of feature reduction, whereas SOM is used in clustering techniques. The PCA technique A is used to speed up feature learning [34]. Before classification, PCA is used by many researchers for feature selection [47]. Anomaly detection is done by using clustering algorithms including k-means and other algorithms based on distance. In NIDS, payload is reduced by using SOM, which is an artificial neural network [7]. In anomaly detection, initial conditions are applied to clustering algorithms, such as the production of a high false positive rate and centroid, so that there is a disadvantage for clustering algorithms [48]. The most important algorithm in unsupervised learning is the k-means. Figure 4 shows commonly used approaches of ML.

#### 3.2.1. Self-Organizing Map

The “Self-Organizing Feature Map” is another name of SOM, and it is the most important neural network model for unsupervised learning. The main purposes of SOM are the clustering of data and reduction of dimensions. Generally, there are two layers of SOM: one is the input layer and the other is the map layer. When data clustering is performed by the SOM, the total neurons of the map layer are equal to the required number of clusters. A weight vector is assigned to each neuron. The problem of data clustering is solved by the SOM algorithm. In [50,51], SOM algorithm is discussed in more detail for interested readers. 

#### 3.2.2. k-Means

Another popular algorithm of unsupervised learning is the k-means method; the main purpose of this technique is the recognition of unlabeled datasets from various clusters. Only two parameters are required for the implementation of the k-means algorithm, which are the initial set of data and required cluster numbers [52]. The problem of node clustering is resolved by following these steps, with k as the required no. of clusters: (1) Randomly select k nodes to initialize k centroids of clusters. (2) Each node is labelled using a distance function with the closest centroid. (3) In accordance with current node memberships, new centroids are adjusted. (4) If the condition of convergence is valid, the algorithm is stopped; otherwise, the process is repeated from step two. In [53,54], the k-means algorithm is explained in full detail.

### 3.3. Semi-Supervised Learning

Unlabeled data is used in this type of supervised learning for training. A small set of labelled data is included in the training dataset, but it mostly consists of unlabeled data. This type of learning is more suitable when large amounts of labelled data are not available, such as when pictures in the archive are mostly unlabeled and only some are labeled [53].

The accuracy of NIDS is enhanced by using SVM of semi-supervised learning [39]. Unknown attacks are detected by the two classification methods of semi-supervised learning: (a) The Gaussian fields approach and (b) Spectral graph transducer. The detection system performance is improved by using the MPCK-means clustering method of semi-supervised learning [55].

An efficient and very easy technique of semi-supervised learning is Pseudo Labelling [56,57]. Basic concept of Pseudo Labelling is also very simple and easy to understand. Initially a model is trained by using a labelled data. Then pseudo model of data which are not labelled is predicted by using the trained model. At the end, model is again trained by using newly pseudo-labelled data and already labelled data. Some other methods of semi-supervised learning (such as expectation maximization, graph-based methods) are discussed in [58].

### 3.4. Reinforcement Learning

Reinforcement learning (RL) based game theory and deep reinforcement learning (DRL) are included in RL. An action support “A” and a representative and state support “S” are involved in RL. The learning entity of RL is the agent; the agent maximizes long-term reward by learning the best actions when interacting with its environment. In RL, the long-term reward of an action is calculated by using a value function in a given state. The “Q-function” is a commonly used value function; the q table is learnt by Q-learning using the Q-function. All pairs of state-actions and corresponding long-term rewards are stored in the q table [59].

#### 3.4.1. Deep Reinforcement Learning

The deep reinforcement learning method is preferred because it does not require prior information regarding the exact model based on the mathematics of the environment. There are some shortcomings in the traditional RL approach, as it is unable to deal with high-dimensional problems and it has a low convergence rate to best performance policy π. DRL can address these shortcomings of the traditional RL approach [60]. The main idea of DRL is approximating the value function by leveraging the deep neural network’s property of function approximation. When deep NNs are trained, they are given an input in the form of a state-action. Then, long-term reward is estimated by DRL. This ultimately helps the agent in the selection of the best action.

#### 3.4.2. RL-Based Game Theory

Premeditated interactions between rational decision-makers are focused on this mathematical tool of game theory. Group of players, utility functions, and techniques are involved in a game [61]. Decision-makers are always players. Optimal strategies are selected by players by using utility functions. Game theory is basically divided into two branches: (a) Cooperative game theory and (b) Non-cooperative game theory [62]. Conversely, players of the second branch play against one another and a utility function is maximized by each player by choosing strategies. The assumption usually made in the network field is that nodes are selfish [63,64,65,66]. The focus of this paper is only on the second branch of game theory. In this branch, communication between players is not allowed and players do not know the selected techniques of the other players at the start of each round. Each player broadcasts their selected strategies (external information) at the end of each round. However, the strategies of other players can affect the utility of each player. In such cases, other players’ strategies should be predicted using methods of adaptive learning, which depends on the optimal strategy for everyone. Optimal strategies by each player can be selected by an adaptive learning method known as RL. RL uses historical information for the selection of strategies, such as status of network, strategies, and utility of other players [67,68,69]. Thus, an effective technique of decision-making is RL-based game theory.

NIDS are developed by using different techniques of machine learning, such as self-organizing map, SVM, RF, ANN, and Naïve Bayesian methods. NIDS was implemented to reduce features in [64], on the basis of RBM, and for problems regarding classification, SVM was used. The approximate accuracy of the system was 87%. The anomaly detection system of the network was developed in [70], on the basis of used discriminative RBM, along with other generative models. It had good accuracy of classification of developed detection was also able to collect data from the training dataset. Prediction of network events is done in [71] by evaluating eight tree-based algorithms of classification. Features are selected by using decision-tree algorithm while classification for NSL-KDD dataset is done by applying random forest algorithm.

In Table 3, we provide a summary of machine learning algorithms, along with the problems they address, and their advantages and disadvantages.

### 3.5. Deep Learning in NID

**Figure 6:**
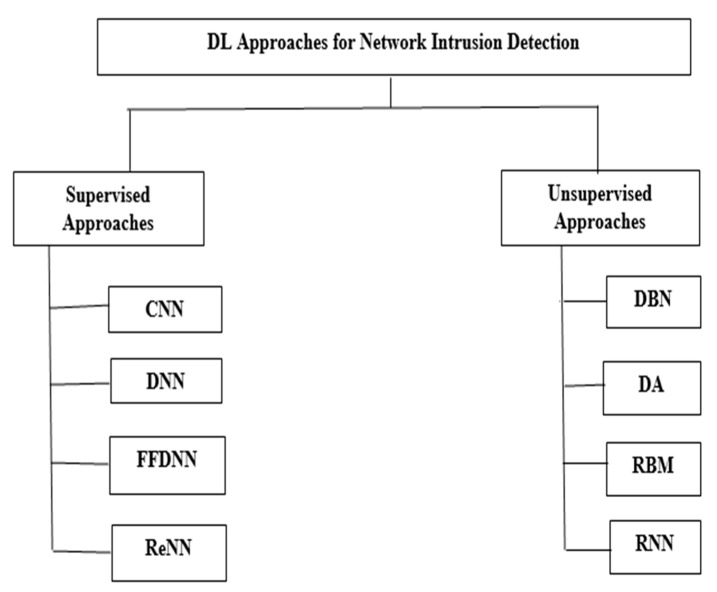
DL approaches used for NID system. RNN: recurrent neural network.

DL methods are advanced types of neural networks that can handle raw bulk data as inputs, meaning it does not require trained input [72]. In DL approaches, there are different kinds of hidden layers in which, with the help of various generalization levels, an algorithm learns the data representation. Normally, the DL algorithm is applied for object detection or recognition, network-based IDS, and many other domains. DL algorithms can be trained in two ways: supervised or unsupervised [73], as shown in Figure 6.

Supervised DL algorithm: This is a learning function where an input and sample-corrected output are given, and the modelling of output from input relies on the sample of corrected output. A function comprising the training example set is extracted from the corrected output, where each example comprises the desired output and input. In DL methods, a class of deep neural networks (DNN) is trained in a supervised way, namely the convolutional neural network (CNN). Normally, CNN can be applied for visual imagery analysis. For structuring 2D images and face recognition, CNN can be used, and for the purpose of computer vision, CNN is now considered a benchmark method [74].

Unsupervised DL algorithm: In this, a model network is pre-trained so that it can be used for other tasks. A type of DL approach called the deep auto encoder (DA) is trained in an unsupervised way; DA is normally used to reduce the dimensionality of a set of data. Another type of DL approach called the deep belief network (DBN) reconstructs input when trained in an unsupervised way and a supervised way of training is used to perform classification [75]. For NIDS, various approaches of DL can be used.

#### 3.5.1. DNN

In [76], SDN based NID system was proposed and in this an algorithm of DL that is DNN was employed. In this study, a NID system was implemented in SDN control plane to monitor all OpenFlow switches. In this proposed NID system, two class classification (anomaly and normal class) was used of the NSL-KDD dataset. There were four categories which dataset contained named as: probe attacks, U2R attack, R2L attack, and DoS attack. The experimental evaluation of this study claims the high accuracy ratio of detection.

In [77], proposed IDS system for vehicular networks in which DNN techniques was used. This method proposed to detect any injected suspicious data packet in the main network controller of automatic vehicles. The proposed scheme maps the learned features on input and differentiate malicious input packets from normal inputs packets and based on this differentiation it classifies the packets into 2 classes: one is attack packet class and second is normal packet class. Further according to activation function, it keeps normal packet class and rule out attack packet class, and the selected normal packet class is sent to next hidden layer and so on. The detection accuracy of this proposed scheme was high with the detection ratio of 99% and the rate of false alarm was very less up to 1–2%.

In [78], IDS was proposed to classify cyber-attacks by using the technique of DNN. In this proposed system there were three main phases named as: acquisition of data, preprocessing of data and DNN classification. This system achieved the accuracy up to 96.3%, the experimental results of this study show that this scheme can give better results as compared to three ML approaches such as k-NN, random forest, and linear regression.

#### 3.5.2. FFDNN

In [79], a system to detect intrusions was proposed by using the DL algorithm FFDNN. This study proposed for wireless networks and in this system selection approach based on feature with FFDNN was used to generate features’ subsets with increased optimization and with reduced redundancy. In this system, the main training data is divided into two subsets: one is evaluation and second is training dataset. Then, it involves the process of two-way normalization and transformation. The used dataset was NSL-KDD, and then finally FFDNN used both for training and testing purposes. The experimental evaluation shows that suggested scheme achieved the detection accuracy up to 99.69%.

#### 3.5.3. RNN

In [80], IDS was proposed based on RNN to perform LSTM and the used dataset was KDD Cup 1999. In this study, as input vector 41 features was used and for output vector 1 non attack and 4 attacks were used. The experimental results shows that accuracy of detecting attacks is 98.8%.

In [81], a system was proposed as anomaly detector in automatic vehicles based on RNN. The LSTM is used as an RNN, in order to forecast new data packet values, LSTM is trained and to detect anomalies the errors of LSTM are used as indicator.

In [82], propose SDN based intrusion detection system by using gated recurrent unit RNN. This study gained 89% of detection accuracy by using reduced number of features. In this scheme the used dataset was NSL-KDD and four parameters depicts its performance named as accuracy, precision, F-measure, and recall.

In [83], multichannel IDS system was proposed by using LSTM based on RNN. For evaluating the performance of this scheme, the used dataset was NSL-KDD. The experimental evaluation shows that performance of this LSTM-RNN was 99.23% with 98.9% accuracy and 9.86% false alarm rate.

#### 3.5.4. Convolutional Neural Network

In [84], an anomaly traffic detection scheme was proposed based on two NN layers. In these two layers, one was comprised of LetNet-5 CNN, and the other consisted of LSTM. For the extraction of spatial features, the first layer was used; the other layer was used to extract temporal features of the flow. For evaluating the performance of this scheme, the CICIDS2017 dataset was used, and the experimental results show that the detection ratio was more than 94%. In this paper, a comparison between some ML algorithms and the proposed scheme was performed, and the experimental evaluation showed that the suggested system was comparatively better than other ML approaches, including random forest, decision tree, logistic regression, and naive Bayes methods, in terms of its F1-measure, recall, precision, and accuracy. Based on these findings, the authors argued that the proposed technique was a more appropriate light-weight framework for intrusion detection, encrypted traffic classification, and detection of novel attacks.

In [85], a network-based IDS was proposed using a convolutional autoencoder, and this study used two datasets: the CTU-UNB and Contagio-CTU-UNB datasets. To build a NN model, a Theano model was used. The experimental results showed that the accuracy rate of this proposed scheme was 99.5%.

#### 3.5.5. Restricted Boltzmann Machine (RBM)

In [86], an IDS system was proposed using a RBM in combination with a deep belief network. In this study the NSL-KDD1999 dataset was used, which consists of 311,029 testing records and 494,021 training records. To implement this detection algorithm, Microsoft Visual Studio 2013 and C++ was used. The experimental results of this study showed that 92% of attacks were classified through RBM.

In [87], a comparative study was presented on using RBM for the detection of intrusions in cyber security. Fundamentally diverse computing models are at the heart of what sets cyber-physical systems (CPS) apart from other kinds of systems. The physical side is governed by the same laws that govern everything else in the universe; differential equations explain system behavior [88]. This study evaluated RBM performance in differentiating between malicious and normal NetFlow traffic. To evaluate the performance of this scheme, the ISCX dataset was used. The experimental results showed that when learning rate was 0.004, the highest accuracy could be achieved, at 78.7 ± 1.9%. Moreover, the true negative and true positive rates were high at a learning rate of 0.004, being 82.4 ± 1.8 and 74.9 ± 4.6%, respectively.

In [89], an IDS system based on stack RBM was described. This scheme was used to detect malicious or anomalous activities. For evaluating the performance of this scheme, NSL-KDD dataset was used, and classification of attacks was done into five classes. In this study for training purposes 40% data was used and for testing purpose 60% data was used. According to experimental evaluation the detection accuracy of this scheme is 97.5%. 

#### 3.5.6. Deep Belief Network

A detection system for intrusions using DBN was proposed in [90]. In this scheme, DBN was used to fabricate FFDNN for IoTs. To reduce the total cost of the IDS model, a cross-entropy binary loss function was proposed by the author. The Keras library was used to sequentially create the DL model. This scheme was designed to detect five different types of attacks: DDoS, opportunistic service, black hole, wormhole, and sinkhole attacks. The Internet of Things (IoT) is an emerging technology that allows objects to quickly and effectively share data across remote networks, such as the cloud or wireless connections. Cyber assaults, which may lead to deadly incursions, are becoming more common due to the changes and advances in the IoT ecosystem [91].

Another study [92] proposed an IDS system using a probabilistic neural network and DBN. To evaluate the performance of this scheme, the authors used the KDD CUP 1999 dataset; 10% of the data was used for testing purposes and another 10% of the data was used for training purposes. The suggested scheme was compared with three other models: optimized DBN with probabilistic NN, PCA with conventional probabilistic NN, and conventional probabilistic NN. The experimental results showed that the proposed scheme was better than the other three models.

#### 3.5.7. Deep Autoencoder

A system to detect intrusions in cyber security was suggested in [93], based on a deep autoencoder. In this study, a DA model was used to improve classification results, which consisted of multiple non-symmetrical hidden layers. The results were as good as the results of the DBN. Two datasets, the NSL-KDD and KDD C’99 datasets, were used to evaluate performance, and five matrices were used in the performance evaluation, i.e., F-score, false alarm, recall, precision, and accuracy. The experimental results showed that, for the KDD C’99 dataset, the average accuracy of the proposed scheme was 97.8%, which exceeded that of other studies of this type. Moreover, the total average accuracy of the proposed scheme for the NSL-KDD dataset was 85.4%, which was a 5% improvement compared with the DBN.

In [94], an IDS system was proposed named TSDL, which was based on the DL algorithm. This proposed model consisted of two stages. A soft-max classifier was used in the TSDL system with a stacked DA; the classifier had three main layers, called the input, hidden, and output layers. The FFDNN was employed as a multi-layer perceptron. For the performance evaluation of this method, the authors used the UNSW-NB15 and KDD C’99 datasets. The experimental results showed that for the KDD C’99 dataset, the recognition rate of the proposed scheme was 99.99%. Moreover, for the UNSW-NB15 dataset, this scheme could achieve up to a 89.12% recognition rate.

The precision of digital measurements is crucial for the monitoring and management of electrical power systems. These digital readings accurately represent the sensors that have been set up, but they are susceptible to the introduction of random variables in the form of hardware failure or malicious hacking [95]. Table 4 shows various DL algorithms for NID systems.

## 4. ML- and DL-Based IDS in SDN

In the following sections, we describe ML- and DL-based approaches used to efficiently detect and prevent intruders in the SDN.

### 4.1. Machine Learning-Based IDS in SDN

In SDN, one of the most significant usage of IDS is to ensure security. As traffic statistics are provided by Open Flow protocol (communication medium between switches and the controller), using messages such as “Stats Request” and “Stats Response”, IDS is the most compelling tool for the identification of threats and anomalies. For both traditional and SDN environments, the operations of IDS are equally applicable.

In [108,109], some examples of IDS using traditional SDN techniques are explained. In [110], anomaly problems were identified leveraging SDN. The main intention of proposing an SDN-based solution was to determine the main problems regarding cloud computing environment security that would react when an attack occurred. On the other hand, the authors in [108] used NFV and NID to create a deep packet inspection system. In [56], the authors used a detection technique based on statistics to get rid of abnormalities in SDN. “Normal profile of traffic” is defined as it is the foundation of statistics analysis. Information based on statistics at the packet level for the network and RMS, such as size of packet and variance, are related to the traffic profile. Traffic of network can be characterized by using the Hurst parameter, H, for instance, to measure bustiness and self-similarity (H is higher when traffic is bustier) [109].

In Table 5, a summary of some surveyed papers is presented. In the framework of ML-based IDS, SDN plays a vital. Details about how SDN is leveraged in the method is provided in the following section [111].

(a) Data Plane

**Figure 7:**
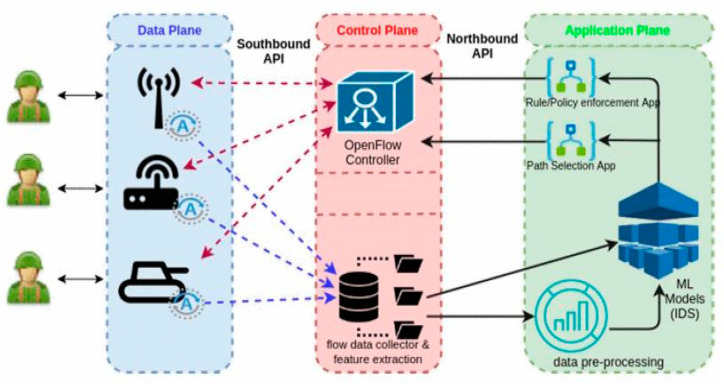
ML-based NID in SDN [46].

In this part, all network devices are immersed of collector agents. As shown in Figure 7, centralized collector records send flow and agent samples. Specific flow metrics are collected by the device configurations, and then they are exported to the collector. Currently, major vendors such as Cisco offer export support and built-in flow collection.

(b) Control Plane

Records of network flow are collected by the data collector embedded in the module of the control plane. Then, the control plane filters the data and conducts feature extraction. Thus, different datasets are created and generated by the collector, which are crucial for the ML approach [46]. The OpenFlow controller should be communicating with all network devices known as data sources.

(c) Application Plane

The constructed and implemented model of ML is used as an application of SDN. Various methods of ML using generated datasets of different flow collectors can be used as applications of SDN for different purposes. The operation of a network can be influenced by constructing different applications powered by the ML-based model. Applications of incident handling include selection of path and rule enforcement. In the following case, the ML model used is an application of SDN-based IDS.

It is predicted that network flows can be classified as being normal or malicious. Figure 7 depicts this process.

#### 4.1.1. DoS, U2R, Probe, and R2L

Four types of attacks are addressed in the studies discussed in the following section: DoS, U2R, probe, and R2L. The NSL-KDD dataset used between all of these include the common characteristics and attacks classified in the four classes.

The D-NN was used by the authors in [5] to detect six features based on anomalies and are suitable for SDN: type of protocol, count, duration, srv count, src bytes, and dst bytes. The model was trained and tested by the authors, and compared with other processes, such as SVM, NB, multi-layer perceptron, J48, random tree (RT), and random forest (RF) methods. The paper explore the applications of DL used in a detection system known as flow-based anomaly detection. At the same time, the authors claimed that the development of machine learning is not fully completed. Ref. [112] presented a discussion of nine classifiers based on ML with a supervised learning technique. Different tests were performed on accuracy, recall, execution time, false alarm rate, area under curve of ROC, f1-measure, McNemar’s test, and precision. PCA was used in the tests to reduce dimensions with DT, K-NN, LDA, NN, linear SVM, extreme learning machine (ELM), BaggingTrees, NB, RUSBoost, RF, AdaBoost, and LogitBoost. The results showed that performance of bagging and boosting techniques was higher than the other techniques. A subset of dataset features were selected as features in which content features were not included. A hybrid classification system of level-5 was used by the same authors in [113] for IDS, in a network that was not based on SDN. Use of flow-statistics was the main aim of the paper; these flow-statistics were provided by the controller for the development of NIDS. In the first level, k-NN was used as the classification method; for the second level, ELM was used. For the other levels, the hierarchical extreme learning machine (H-ELM) was used. One type of attack was detected by each level using the same preferred features from [113]. For scalability purposes, the implemented system was in the form of a POX controller module, rather than an application plane function. The effectiveness of the method chosen to select features was because the features could be directly accessed from the controller. The results showed improved accuracy compared with the other methods.

#### 4.1.2. DDoS Attacks

In the following section, DDoS attacks are specifically investigated in the presented studies for two reasons. First, DDoS attacks have been focused on large sections of IDS studies. Second, attacks should be individually considered with the perspective of recent threats, for example, the Mirai botnet and Internet of Things (IoT) [114]. A specific application using SDN was presented by the authors in [115] to tackle challenges in anomaly detection regarding scalability. The scenario was wireless SDN, which enabled the E-Health system. Massive machine-type communications were the main feature of such a network, where humans do not interact. For semi-supervised operations, CPLE was the ML technique used, with offline training. The main intention was online testing, so running localized detection was allowed within the devices. The requirement for frequent network traffic collection was avoided by using online testing to update the model of anomaly detection. The features used for classification were similar to the features defined in [116]. An overview of IDS based on ML in SDN is provided by authors in [112].

Millions of people may experience significant power outages if an attacker exploits cyber security weaknesses. This study addresses this problem by presenting an OPNET-based network model exposed to many DoS assaults, illustrating the cyber security features of IEC 61850-based digital substations [117].

Five ML techniques were investigated by the study to mitigate DDoS attacks and intrusion (support vector machine, neural networks, Bayesian networks, genetic algorithms, decision tree, and fuzzy logic). Each method was theoretically analyzed by authors and a comparison scheme was generated that presented the advantages and disadvantages of the approaches. Our review could be used to choose the best technique, in accordance with system requirements. In [118], the authors compared the SVM analysis in SDN with other approaches in defending against a DDoS attack. Threats to the controller regarding security and types of SDN-based DDoS attacks are briefly discussed in the paper. Moreover, four methods of SVM and a description of system was provided in the paper. For training and testing, the datasets used were the 1999 and 1998 DARPA, and a comparison of approach was done with bagging, RBF, random forest, J48, and naive Bayes methods. Highest accuracy was shown for the proposed SMV, at approximately 95%. The support vector classifier-based learning algorithm, where features are selected by using an ID3 decision tree, was used by authors in [119]. The following three components, along with the software testbed, was used to evaluate the model: (1) Data collection was done using the sFlow Toolkit. (2) For the virtual switch, Open vSwitch was used. (3) The controller used was Ryu and the dataset was KDD-Cup 1999.

The model used in [120] was the Dirichlet process mixture model, to mitigate DDoS attacks based on DNS. An owned dataset was used by the authors in [3]; they created a dataset to generate DDoS attacks. The IDS system was presented by the authors in [121] for the identification of DDoS attacks. They compared three methods: SVM with 97% accuracy, KNN BEST was 83% accurate, and naïve Bayes was approximately 83% accurate. Features considered as inputs included the number of packets, bandwidth, destination IP, protocol, source IP, and protocol. They used an owned dataset for testing. A proposal was presented by the authors of [24] to improve resiliency by detecting some DDoS attacks, preferably the SYN flood attack, in the SDN network. Three different techniques were studied for classification: NB, DT, and SVM. DT showed 99% recall, precision, and accuracy. KDD 99 was the dataset used, with features including protocol, src port, src IP address, dst port, and dst IP address. Later, PCA was used by the authors for reduction. DDoS attack detection and classification was done by authors using an approach in the cloud environment [122]. They used a two-stage learning scheme with two stages using two techniques: Bayesian and multivariate Gaussian. The employed features were blacklist IP, dst IP, number of packets, src IP, and spoof dst IP. Although complementary elements to the ML method were included in the study, however they did not directly secure the SDN. As an alternative, some steps were for the security of cloud infrastructure. 

#### 4.1.3. Comparison of Various Approaches in SDN

When considering a wide range of attacks regarding cyber security, it is important to have five attacks, including DDoS, U2R, DoS, probe, and R2L. Though SDN is an innovative paradigm, it is still subject to all known attack types. New adapted attacks should also be considered by the research community. Reviewing the adaption of approaches to SDN is essential for the recognition, prevention, and extenuation of attacks. In all traditional networks, the main point of applied ML approaches are to recognize attacks. ML uses miscellaneous techniques. Most of the studies we reviewed investigated a single ML approach. One of two approaches from at least two methods was used in other papers; the techniques were compared or combined to improve anomaly detection. Half of the reviewed papers used neural networks (generic NN, CNN, RBM, ANN, and NEAT), as shown in Table 5 and Table 6. SVM was another approach used in the reviewed papers. The naïve Bayes method was also presented in several articles. However, a set of six features suitable for SDN was presented by authors in [123], which were further used in four studies. In different cases, independent selection of techniques of features are conducted, which have to be included in ML. In [124], it is demonstrated that a network attack in SDN can be predicted with an accuracy of 91.68% using Bayesian Networks machine learning approach. 

**Table 5:** Different ML techniques for NID in SDN with training datasets.

Reference	Method of Detection	Dataset Used	Detected Attack	Feature Selection
[122]	RBM	KDD-Cup 1999	General anomaly	41 features
[114]	Random forest	KDD99	DoS, R2L, U2R, and Probe	10 feature sets
[125]	SVM	NSL-KDD	DOS	25 used from 41 features
[126]	k-means	Simulation-based	UDP flood and TCP flood	Packet count, duration, and byte count

### 4.2. Deep Learning-Based IDS in SDN

There are several studies that have used DL-based IDS in SDN. Normally, seven different kinds of threat vectors exist in the SDN, of which three threat vectors are definite and linked with the controller application plane, controller data plane, and control plane. NIDS is broadly used for detecting intruders within the network by continuously monitoring any suspicious and malicious behavior in network traffic. Based on strategies for network attack detection, NIDS are mainly of two types. The first strategy compares network traffic with pre-defined intrusion samples, and this is called signature-based detection [129]. New kinds of attack strategies cannot be detected by this kind of NID system; despite this fact, this technique is still very popular and commonly used in commercial IDS. In the second strategy, network traffic is compared with a normal user behavior model and any deviations from normal behavior of traffic is marked as an anomaly, using ML approaches. This type of NIDS is called anomaly-based detection. This technique can even detect attacks that have never seen before. Normally, flow-based monitoring of network traffic is combined with the latter NIDS strategy, i.e., anomaly-based detection [130].

Flow-based network traffic monitoring relies upon packet header information, which is why it handles a lower amount of data compared with payload-based NIDs,. Applications of ML approaches are found in multiple zones of computer science, including speech recognition, face detection, and intrusion detection systems; however, such ML applications have faced some issues. In [131], the authors discuss the various issues in which ML algorithm applications affect the NID system. Although deep learning research on NID systems that leverage SDN is in its pre-stages, it is gaining more attention among researchers due to its results. Until now, DL algorithms have been extensively used in different areas of computer science for image, face, and voice recognition and has had real success. Through DL algorithms, correlations in bulk amounts of raw data can be easily found, and due to this feature, it can be broadly used in the next generation of NID systems. With the help of DL-based NID systems, one can obtain high detection accuracy and even efficiently detect attacks that have never been seen. In another study [132] authors investigate how DL might be useful for detecting malicious java script code.

There are many advantages exhibited by the SDN-based NID system using deep learning algorithms, including quality of service, virtual management, and security enforcement. SDN eliminates dependency on hardware because the whole network can be configured through programming and SDN enables a global view of the entire network, providing the chance to strengthen the network security [133]. In Table 7, different SDN-based NID systems using a deep learning algorithm are briefly overviewed and compared. Using emulation and simulation platforms, SDN can be developed with programmable features and software switch implementations. SDN can be easily implemented in both software and hardware environments with the help of protocol standards, i.e., OpenFlow [134]. OMNeT++ [135], NS-3, Mininet [136], and NS-2 are some other simulation tools used for SDN. In SDN, its control plane is considered its most vital part, and is also called the operating system of the entire network. The control plane of SDN is accountable for providing a global view of the entire network and communicate with all programmable network elements. Beacon, OpenDayLight [137], Ryu, Floodlight [20], POX, and NOX [21] are different kinds of controllers.

The focus of recent studies have been on employing DL algorithms in NIDS, rather than ML algorithms. Through DL algorithms, correlations in bulk amounts of raw data can be easily found, and due to this feature, it could be broadly used in the next generation of NID systems. Compared with the results of various NID systems based on ML algorithms, DL-based NID systems gave much better results in the context of SDN [22]. Most machine learning algorithms are trained in a supervised way and these can give good results in classification tasks, but not in logic modelling; deep learning-based algorithms outperformed ML algorithms in logic modelling. As attack behaviors consistently change, they introduce new types of attacks, and unsupervised learning approaches such as RNN, stacked deep auto-encoder, and hybrid approaches are the best options in detecting these attacks in SDN-based NID systems. Currently, researchers are focusing on SDN-based NIDS using DL algorithms for SOHO networks and their satisfactory results suggest that intrusion detection system accuracy has greatly improved because of SDN scalability and DL algorithms [138].

#### 4.2.1. DDoS Attack Detection Using DL Algorithms

Different kinds of vulnerabilities exist in the SDN platform, due to which the architecture of SDN is being targeted by various kinds of attacks such as a DDoS attack. In a DDoS attack, the intended user cannot get access to the network resources or machine. Multiple bots or multiple people are usually responsible for a DDoS attack, e.g., to launch a DDoS attack, the intruder can take advantage of SDN characteristics against the application plane, infrastructure plane, and controller of SDN. In the SDN environment, it is very easy to deploy a DDoS attack; preventing such an attack is very difficult [139,140]. The occurrence of DDoS attacks in SDN is increasing daily, since the advent of the internet. The major reason behind the increased occurrence of DDoS attacks is the development and emergence of botnets that are formed within a network by machines or bots when they are exploited with malware. According to [141], the increase in DDoS attacks in 2016 was 125.36% of those that occurred in 2015. In [142], a lightweight detection system for DDoS attacks based on SOM in SDN was suggested, with a 6-tuple feature extraction: growth of different ports and single-flows, percentage of air-flows, average of duration per flow and bytes per flow, and packets per flow. In the flow-table, statistics features are extracted at certain intervals and are used in the implementation of this proposed method, making it a light-weight system. However, as a downside, this system has some limitations; it cannot be used for traffic handling that is not based on flow rules. Enhanced power plant monitoring is now possible thanks to modern sensors. As part of the overall cogeneration procedure, cooling towers condense exhaust steam to cool the facility [143].

In [144], an SDN-based DDoS blocking application was designed that could block a DDoS attack. For attack detection, this scheme worked in cooperation with two targeted servers. This was a prototype study that was designed to detect HTTP flooding attacks. In [145], the authors proposed a technique to detect a DDoS attack by using entropy calculations in the SDN controller and a deep auto-encoder approach for feature reduction. To detect attack, a threshold value was implemented, and selection of the threshold value was based on experimental results. A downside to a vast network is that there is a controller bottleneck; it can also affect the reliability affects because the threshold value can change in different scenarios.

In [146], a system combining a fuzzy interface, a hard detection threshold under attack, and normal states based on real characteristics of traffic was proposed for the detection of DDoS attack. For attack detection, three features were chosen, including flow quality to a server, packet quantity distribution per flow, and interval time distribution. Currently, researchers are working on the detection of flow-based intrusion.

In [112], IDS was also placed by the authors in the control plane. NSLKDD was the used dataset and a meta-heuristic Bayesian network was the technique used for the classification of traffic. Phase of selection and extraction of features was included in the proposed process to optimize the classifier. Fitness evaluation of the features which were selected was included in the classifier. After that, a Bayesian classifier was used. Seven other approaches are used were the comparison, but this proposed method was more accurate than the other algorithms, having 82.99% accuracy.

#### 4.2.2. Anomaly Detection Using DL Algorithms

In the SDN environment, many approaches have been implemented for anomaly detection to secure the OpenFlow network. In [147], the author designed a programmable router for a home network by using the programmability feature of the SDN network to provide an ideal location and platform for detecting malicious behavior in SOHO (small office/home office). The four most popular SDN-based anomaly detection methods include NETAD, maximum entropy detector, rate-limiting, and TRW-CB algorithms were implemented in NOX and the OpenFlow compliant switch of SDN. Experimental results of these algorithms showed that, in detecting malicious activities, these algorithms had more accurate results in SOHO networks compared with ISP. Without introducing any new performances overhead, these anomaly detection algorithm work at line rates for the SOHO network.

In [148], an anomaly-based detection system based on flow was proposed, using a gravitational search algorithm and multi-layer perceptron. Experimental results showed that this proposed system gave a high accuracy ratio in classifying malicious and benign flows. In [149], an SDN-based NID system was proposed by using SVM. Experimental results showed that the positive alarm rate was very high, with a lower false alarm rate. In this study, the traffic system was trained with malicious network traffic, rather than with normal data.

In [150], the authors proposed an anomaly detection system based on DL algorithms in which flow for anomaly detection and OpenFlow was combined to reduce overhead processing. In this proposed method, the false positive rate was high in detecting attacks because network traffic flow was used for implementation.

The network traffic of social multimedia is continuously increasing due to increases in usage and continuous development of multimedia services and applications. The secure transmission of data requires a network that includes features of quality of service, quality of information, scalability, and reliability. In this context, SDN is a significant network, but energy-aware networking and runtime security affects its capability; thus, to increase SDN reliability, a SDN-based anomaly detection system was proposed in [1]. By using DL approaches in the context of social multi-media, this system was used to detect any suspicious flow in network traffic. This proposed system consisted of two modules. The first was an anomaly-detection module based on RBM and gradient descent-based SVM for the detection of any suspicious behavior. The second module was end-to-end data delivery to satisfy SDN’s quality of service requirements. For the performance evaluation of this proposed scheme, both benchmark and real-time datasets were used. Experimental evaluation showed that this proposed scheme was very efficient and effective in effective in data delivery and anomaly detection for social multimedia.

Some studies used deep learning for general anomaly detection. In the paper [151], the authors presented DL-based IDS for SDN environments. IDS was implemented as a component of the control plane in both studies, rather than using it as an application. The location allowed for direct interaction to protect the controller. Moving target defense and IDS was the aim of the authors [151]. To get data from training, a simulated network was generated by the authors (of about 40,000 packets). A neuroevolutionary model was presented as a light-weight detector for the architecture, by which real-time operation was allowed. Two different detectors, DDoS and worm, were developed to achieve it, with each detector identifying one type of attack. “Neuroevolutionary of Augmenting Topologies (NEAT)” was used by authors to combine the two detectors. NEAT is a method related to neuro-evolution with a crossover background.

The general environment of SDN was presented by authors in [4] with unsupervised learning. The auto encoder was used in the approach; the encoder and decoder were the two phases of the auto encoder, used to identify reconstruction error and minimize it for each test sample. TensorFlow was the development library, but the used dataset was not clear.

#### 4.2.3. Specific Circumstances of Network

Some studies also investigated specific circumstances of network. The implementation of IDS based on DL in optimal SDN was presented in [127]. Attacks were reviewed in the control plane in [128], and then they were classified into leakage of data, modification of data, unauthorized access, misuse of security policy, and denial of service. Anomaly detection considered features about optimal links, as the process was based on optical networks. These included usage of average bandwidth, destination nodes, formats of modulation, frequent source, and average length of route. Light-path creation, deletion, and modification were some attacks included in related networks. Point-anomaly-based methods were the first detection methods, with a point being used to represent a data instance. Probability was calculated by a user-created algorithm. A sequence-anomaly based method was the second detection method, where the occurrence of anomalies was in a sequence and a cumulative sum approach was used. NSFNET topology was used by the authors to test an owned dataset, and the results of the detection method showed 85% accuracy.

Numerous compromised nodes were included in the attack by which synchronized traffic with low intensity was generated to disconnect hosts and links from any network. There is a growing reliance on digital measurements in the monitoring and managing of electrical power networks. Recently, wide-area monitoring systems (WAMS) have been established to enhance the situational awareness of complex networks and, by extension, their transmission efficiency [152].

Coordinated attacks are classified using three DL techniques including convolutional neural networks, artificial neural networks, and LSTM networks. In [128], a testbed was created by authors for the generation of their dataset in Mininet [153] with increased traffic. After that, training and testing of the model was performed using this dataset. The results showed that when there was an increase in the speed of the vehicles, the performance was reduced, as well as efficiency. The training time of each algorithm was 100 s. The short time allowed the system to re-train as necessary.

**Table 7:** Comparison of NID system leveraging SDN based on deep learning approach.

Paper	Objective	Controller Used	Method	Comparison
[1]	Lightweight DDoS Flooding Attack	OpenFlow Controller	Used SOM with artificial neural network	It could efficiently detect DDoS attack but there were no flow rules installed for detection
[150]	Anomaly Detection	SDN Controller	Used DL approach for detection of flow-based anomaly	Did not have any alternative solutions for signature-based intrusion detection system
[144]	DDoS Attack Detection	OpenFlow Controller (NOX)	Used deep auto-encoder approach for feature reduction	For vast networks, there is a controller bottleneck
[146]	Intrusion Detection	OpenFlow Controller	Used learning vector quantization and SOM	Cannot efficiently detect U2R attack
[148]	Intrusion Detection	SDN Controller	Used DL with generative adversarial networks	It is very efficient and cost effective in intrusion detection
[147]	Anomaly Detection	NOX and OpenFlow Compliant Switches	Used four anomaly algorithms: TRW-CB algorithm, NETAD, maximum entropy detector, and rate limiting	Able to detect anomalies in SOHO network and have standardized programmability
[154]	Anomaly Detection	SDN Controller	DL-based RBM and gradient descent-based SVM anomaly detection for suspicious flow detection	Effective data delivery is realized using multi-objective flow routing scheme based on SDN
[155]	DDoS Attack Detection	SDN Controller	Generative adversarial network-based adversarial training in SDN	Able to continuously monitor network traffic using IP flow analysis and enable anomaly detection in near real-time, used dataset was CICDoS2019

## 5. Discussion

We have discussed SDN-based ML and DL approaches for intrusion detection. SDN-based IDS using ML and DL algorithms showed many advantages, including quality of service (QoS), virtual management, etc. As SDN allows us to create an ecosystem of controlling software and opening switches, we can achieve the innovative and rapid integration of desired operations. Through SDN, the monitoring and processing of data has become easier, and has provided flexibility to program the network to strengthen network security. Therefore, researchers are focusing on SDN for implementation of DL and ML approaches in order to detect intrusions.

Detection of sophisticated attacks is difficult using classical ML techniques for large-scale network environments. Poor classification can be caused by several challenges, such as less available labeled training data and irrelevant features. As shown in Table 3, we compared various ML algorithms and described the advantages and disadvantages for each approach. Detection of attacks via classical ML methods can be problematic due to these factors, as well as the use of obsolete datasets, i.e., NSLKDD and KDD99. These sets of data contain either outdated signatures of network attacks or do not consider essential features of modern traffic [155]

One of the most noteworthy solutions to overcome the weaknesses of ML was the DL method [156]. DL is broadly used by different companies, including Facebook, Google, and Microsoft. They use DL in various applications, such as image processing, translation, and speech recognition. However, the literature shows that there are also some concerns regarding DL approaches for IDS that require more research. Some concerns regarding DL approaches are that they require larger amount of data to perform better, compared with other techniques, and use more complex operation computational time than ML approaches. For example, in the case of the CNN DL approach, detection of intrusions in real time is difficult due to the complex convolution operation [155].

To overcome this issue, feature selection is used so that the space covered by features are reduced and only the most significant features are adopted. Moreover, straight application of ML is not feasible for network traffic analysis due to the huge amount of traffic data. Hence, pre-processing (feature selection) of network traffic plays a crucial role om devising accurate detection techniques. Feature selection methods have been compared for detections of intrusion by authors in [157,158,159,160], also outlining disadvantages of feature selection in the different learning systems (i.e., complexity and expensive implementation). In recent years, feature selection has been an active topic of research, where different methods of feature selection are being used, such as random forest for the reduction of features and feature selection approaches based on bi-layer behavior and principal feature analysis [161,162]. Support vector data description was used for anomaly detection in [163], for the automated selection of optimal feature combinations by applying different techniques of feature selection. Researchers are now interested in using processes of DL, such as RBM, recurrent NN, deep auto encoders, and deep belief network, for reducing the dimensionality of the dataset and selecting appropriate features. DL has also gained popularity in the field of feature selection. In IDS development, different models of DL have been utilized, as high-level features can be represented by them into more intellectual features of data. Selection of appropriate features was attempted by different studies for detection of intrusions [14,91]. Intensive features were automatically extracted by DL approaches from records of data that were unlabeled. Therefore, many tasks regarding cyber security use DL, including analysis of traffic, intrusion detection, etc. Large-scale network traffic can be addressed by DL using different layers of processing to construct the unknown structure in the distribution of input. DL is able to form good representations of input data that traditional methods were unable to do.

## 6. Research Challenges

Some challenges in the development of an efficient and flexible SDN-based intrusion detection in a network, using approaches of ML/DL [164], are listed as follows:

- The accuracy ratio of DL approaches is higher compared with ML approaches for intrusion detection. Unfortunately, accuracy comes at the expense of the time complexity issue due to the complex operations involved. For detecting an attack in real time, extensive research is required on DL approaches [155].
- Selecting an appropriate method for the selection of features is a predominant challenge by which redundancy between selected features and significance of features to the task of NID can be precisely determined. Therefore, improvement in computational realism and evaluation of the optimum no. of model parameters is a huge challenge for both ML and DL techniques [2].
- Appropriate methodologies for assessment and metrics is absent, and comparison of alternative techniques and evaluation of IDS is not possible due to the absence of a general framework. Deep analysis was conducted later, as this issue was very significant.
- For academic research, the accuracy of the existing dataset of intrusion detection is not suitable for prediction of research, as proper data classification is required by them. Synthetic datasets are used by network researchers for detection of intrusions in the network due to the lack of more accurate and realistic datasets. Datasets used for intrusion detection systems, e.g., NSL-KDD and KDD99, are outdated. KDD Cup 1999 is the most common dataset used to evaluate intrusion detection; NSL-KDD, which is the modified form of this dataset is used in IDS systems. It is very important to evaluate systems of network intrusion accurately and consistently by creating datasets [165]. New sets of data CSE-CIC-2018 are available for testing and evaluating intrusion detection; however, more research is required on these datasets.
- It was reported in [166] that attacks can easily affect most systems of intrusion detection, as their dependence power is poor. Descriptions of how IDS is eluded by different mechanisms is given in the literature [167]; the technology of intrusion detection needs to be improved in this aspect. Similarly, DDoS attack in SDN enabled cloud computing environment is also an active research area as discussed in [168].
- One of the most fundamental challenges from NIDS based on SDN is the efficient handling of packet processing flows because the implementation of NIDS using different approaches of ML and DL is significantly affected by this challenge with its high volumes of data.
- Different attacks (e.g., DDoS) may affect the software-defined network. In SDN, some basic potential vectors of threat include attacks on the control plane, forged traffic flows, and susceptibilities in switches. Devastating impact can be caused by all of these attacks on the overall network [6]. Thus, improvement in the security of SDN is required.
- For large networks, a performance bottleneck could be faced by controllers of the network applying SDN because of the large amounts of data (incoming and forwarding). Another big research challenge is to reduce this performance bottleneck of the controller, so that NIDS can be implemented [169].
- Usually, high data rates cause high costs and low throughput by which current wide-band transmission technologies can be characterized [155]. Optimization of intrusion detection is related to techniques of grid and paradigms of distributed detection.

## 7. Conclusions and Future Work

In this paper, various approaches based on the classical techniques of ML and DL were discussed in detail, by which attacks were detected in SDN. The paper also included explanation about the weaknesses of traditional methods based on ML and their poor performance. It was emphasized that vulnerabilities are detected and the network is monitored using approaches of ML/DL in a platform known as SDN. In SDN, specific methods are needed to define data by classification into frameworks and techniques to implement ML approaches. The need for updated datasets was identified, as the latest attacks are used to learn models. Based on this study, the usage of methodologies based on DL were shown to improve NIDS effectiveness and performance in terms of FAR reduction and accuracy of detection. DL approaches were used in about 80% of the proposed solutions with AE and DNN, and these algorithms were used frequently.

It is worth noting that the high speed infrastructure and critical infrastructure of the network did not use any approaches where SDN-based NIDS was implemented. In this area, the future research scope should be to propose an efficient framework of NIDS using fewer complex DL algorithms for a more effective detection mechanism. This knowledge could be used to design an effective, lightweight, and innovative NIDS-based DL. Moreover, intruders could be effectively detected by using these within the network.

## Figures and Tables

**Table 1:** Comparison of current study with similar previously published articles.

Review Paper	Year	Review NIDS	SDN Focused	Include ML	Include DL	Contribution	Limitation
[8]	2017	Yes	No	Yes	No	This research classified IDS. IDS complexity was classified. Compared shallow and deep network learning techniques. Experiments showed deeper networks spot threats better.	Signature-based techniques were used; however, they could not detect all forms of assault, particularly if the IDS signature list was missing the proper signature.
[10]	2018	Yes	Yes	Yes	No		
[5]	2019	Yes	Yes	Yes	No	ML techniques and IDS frameworks for SDN were examined. The second category included data collecting and mitigation strategies. Standard datasets, testbeds, and research tools were included.	The authors provided an overview of obstacles and potential of employing ML with emerging technologies such as SDN; however, it was not thorough.
[7]	2019	Yes	Yes	Yes	No	This paper looked at ML techniques using SDN to generate NIDS. Deep learning was used in SDN-based NIDS. This research examined SDN NIDS modelling tools.	When it comes to network-based IDS, sensors are deliberately deployed around the network to pick up on reconnaissance assaults.
[9]	2019	Yes	Yes	Yes	No	In this overview, machine learning methods helped SDN-based NIDS. This survey included NIDS model-building tools. The two strategies used in this study may enhance network intrusion detection.	Acquiring a new management tool and providing everyone with the necessary instruction is a priority. The lack of security is a major obstacle for the SDN.
[11]	2019	Yes	No	No	Yes	Anomaly-detection applications were evaluated. Categorized deep anomaly detection systems.	Robust features need manual extraction. The frequency of insurance fraud is significantly smaller than the number of claims, and each scam is unique. Such techniques cannot identify fresh failure signals.
[12]	2020	Yes	No	No	Yes	Categorized deep learning-based IDS by input data, detection, deployment, and assessment methodologies. This survey compared and examined deep learning-based IDS experiments.	Due to limited datasets, IDS systems lack real-world dependability and applicability. Benchmark datasets are not real-time.
[13]	2020	Yes	No	Yes	Yes	This article described IDS and proposed a taxonomy for ML and DL-based network-based IDS (NIDS) systems. The examined articles described IDS classification systems.	In general, ANNs tend to overfit to their training data. Due to the iterative nature of selecting the size and structure of an ANN, overfitting occurs all too often.
This Article	2022	Yes	Yes	Yes	Yes	ML/DL algorithms on the SDN platform could find and resolve system problems and monitor the whole network. To utilize ML tactics in SDN, we categorized ML frameworks and methods.	

**Table 3:** Various ML algorithms, the type of problem they address, and their advantages and disadvantages.

Machine Learning Approach	Type of Problem	Advantages	Disadvantages
Random forest	Regression and classification	Instability is reduced. Overfitting of DT model is mitigated. Accurate for huge training sets.	Does not give accurate results for imbalanced training datasets. Training speed is low.
Support vector machine	Regression and classification	High-dimensional datasets can be effectively handled. Valid for both separable datasets (linear and non-linear).	Training of large datasets is difficult. Not good for noisier datasets.
K-nearest neighbor	Regression and classification	Implementation is easy. Flexible.	It is memory-intensive. Computationally expensive.
Self-organizing map	Clustering	Understanding of data mapping is easy. High-dimensional datasets can be effectively handled.	For large maps, it is computationally expensive.
K-means	Clustering	Clustering results can be easily interpreted. Implementation is easy.	Linear computational cost. Sensitive to first outliers.
Semi-supervised learning	Clustering, regression, and classification	Labeled and unlabeled data are used.	Fully depends on assumptions, such as smoothness assumptions and manifold.
Reinforcement learning	Decision-making	Fast decision-making after training. Prior knowledge is not required to work properly.	High-dimensional problems cannot be handled. Low convergence rate
Deep reinforcement learning	Decision-making		More computational resources are required to train datasets.

**Table 4:** DL algorithms for NID systems.

Deep Learning Approach	Model Detail	Dataset	Ref.
DNN	Using DNN for SDN-based IDS	NSL-KDD	[76]
	Using DNN to handle huge data for large network	NSL-KDD	[77]
	Using DNN for intrusion detection system in vehicular networks	Vehicular network communication	[78]
	Using DNN for network intrusion detection system to classify cyber attacks	PROBING, U2R, R2L, and DoS	[96]
	Using DNN to detect privacy attacks and DoS in ad hoc networks	KDD C’99	[97]
	Using DNN to detect network intrusions	KDD C’99	[98]
	Using DNN to evolve network attacks	KDD C’99	[99]
FFDNN	Using FFDNN to detect network intrusions	NSL-KDD	[79]
RNN	Using RNN to detect network intrusions	KDD C’99	[80]
	Using RNN to detect attack against vehicle	Attacks against vehicles	[81]
	Using RNN to detect network intrusions	NSL-KDD	[77]
	Using RNN for intrusion detection system in SDN	NSL-KDD	[82]
	Using RNN for multi-channel intrusion detection system	NSL-KDD	[83]
CNN	Using CNN to detect network intrusions	UMASS dataset	[100]
	Using CNN to anomaly traffic detection	CICIDS2017	[84]
	Using CNN for intrusion detection, encrypted traffic classification, and detection of novel attacks	ISCX 2012 IDS	[85]
	Using CNN to evaluate network intrusions	Contagio-CTU-UNB	[101]
**Machine Learning Approach**	**Type of Problem**	**Advantages**	**Disadvantages**
RBM	Using RBM to evaluate network intrusions	KDD C’99	[86]
	Using RBM to detect cyber security intrusions	ISCX dataset	[87]
	Using RBM for intrusion recognition domain	KDD C’99	[102]
	Using RBM to detect anomalous activities	NSL-KDD	[66]
	Using RBM to traffic detection	Real online network traffic	[103]
	Using RBM for clustered intrusion IDS in wireless sensor networks	KDD C’99	[104]
DBN	Using DBN for intrusion detection in IoT	IoT simulation dataset	[90]
	Using DBN and probabilistic neural network for IDS	KDD Cup 1999	[92]
	Using DBN for cyber security intrusion detection	NSL-KDD	[105]
	Using DBN for IDS in SCADA	IEEE 118-bus and 300-bus	[96]
DA	Using DA for cyber security intrusion detection	NSL-KDD	[93]
	Using DA in IDS	UNSW-NB15 and KDD C’99	[94]
	Using DA autonomous and self-adaptive misuse IDS	NSL-KDD	[106]
	Using DA for cyber security intrusion detection	KDD C’99	[107]

**Table 6:** Different DL techniques for NID in SDN with training datasets.

Reference	Method of Detection	Dataset Used	Detected Attack	Feature Selection
[112]	NEAT	Owned: 800000+Packets	DDoS and worm	3-packet-level features
[127]	ANN, LSTM, and CNN	Owned	Crossfire	3-flow-based features
[128]	DT, NB, and SVM	KDD-Cup 1999	DDoS	4-flow-based features
[4]	MHBNC	NSL-KDD	DoS, R2L, U2R, and probe	Extraction of features and pre-processing

## Abbreviations

**Notations**	**Descriptions**
AI	Artificial Intelligence
AP	Application Plane
API	Application Programming Interface
BMU	Best Matching Unit
CP	Control Plane
CNN	Convolutional Neural Network
CPS	Cyber-Physical Systems
DA	Deep Autoencoder
DL, TLS	Deep Learning, Transport Layer Security
DP	Data Plane
DBN	Deep Belief Network
DNN	Deep Neural Networks
DRL	Deep Reinforcement Learning
DoS	Denial of Service
DDoS	Distributed Denial of Service
ELM	Extreme Learning Machine
FFDNN	Feature Fusion Depth Neural Network
GPUs	Graphics Processor Units
HIDS	Host-based Intrusion Detection Systems
H-ELM	Hierarchical Extreme Learning Machine
IoT	Internet of Things
k-NN	k-Nearest Neighbors
LSTM	Long Short-Term Memory
ML	Machine Learning
NBI	North Bound Interface
NIDS	Network Intrusion Detection Systems
PCA	Principle Component Analysis
RF	Random Forest
RL	Reinforcement Learning
RT	Random Tree
RBF	Radial Based Function
RBM	Restricted Boltzmann Machine
RNN	Recurrent Neural Network
SBI	South Bound Interface
SDN	Software Defined Network
SVM	Support Vector Machine
SOM	Self-Organizing Map
SCADA	Supervisory Control and Data Acquisition System
SOHO	Small Office/Home Office
QoS	Quality of Service

## References

[1] Mehdi S.A., Khalid J., Khayam S.A. (2011). Revisiting Traffic Anomaly Detection Using Software Defined Networking. International Workshop on Recent Advances in Intrusion Detection.

[2] Garcia-Teodoro P., Diaz-Verdejo J., Maciá-Fernández G., Vázquez E. (2009). Anomaly-Based Network Intrusion Detection: Techniques, Systems and Challenges. Comput. Secur..

[3] Ahmed M.E., Kim H., Park M. Mitigating DNS Query-Based DDoS Attacks with Machine Learning on Software-Defined Networking. Proceedings of the MILCOM 2017–2017 IEEE Military Communications Conference (MILCOM).

[4] Dawoud A., Shahristani S., Raun C. (2018). Deep Learning and Software-Defined Networks: Towards Secure IoT Architecture. Internet Things.

[5] Herrera A., Camargo J.E. (2019). A Survey on Machine Learning Applications for Software Defined Network Security. Proceedings of the International Conference on Applied Cryptography and Network Security.

[6] Hu F., Hao Q., Bao K. (2014). A Survey on Software-Defined Network and Openflow: From Concept to Implementation. IEEE Commun. Surv. Tutor..

[7] Sultana N., Chilamkurti N., Peng W., Alhadad R. (2019). Survey on SDN Based Network Intrusion Detection System Using Machine Learning Approaches. Peer-Peer Netw. Appl..

[8] Hodo E., Bellekens X., Hamilton A., Tachtatzis C., Atkinson R. (2017). Shallow and Deep Networks Intrusion Detection System: A Taxonomy and Survey. arXiv.

[9] Tiwari S., Pandita V., Sharma S., Dhande V., Bendale S. (2019). Survey on Sdn Based Network Intrusion Detection System Using Machine Learning Framework. IRJET.

[10] Xie J., Richard Y.F., Huang T., Xie R., Liu J., Wang C., Liu Y. (2018). A Survey of Machine Learning Techniques Applied to Software Defined Networking (SDN): Research Issues and Challenges. IEEE Commun. Surv. Tutor..

[11] Chalapathy R., Chawla S. (2019). Deep Learning for Anomaly Detection: A Survey. arXiv.

[12] Aldweesh A., Derhab A., Emam A.Z. (2020). Deep Learning Approaches for Anomaly-Based Intrusion Detection Systems: A Survey, Taxonomy, and Open Issues. Knowl.-Based Syst..

[13] Ahmad Z., Khan S., Shiang W., Abdullah J., Ahmad F. (2021). Network Intrusion Detection System: A Systematic Study of Machine Learning and Deep Learning Approaches. Trans. Emerg. Telecommun. Technol..

[14] Injadat M., Moubayed A., Nassif A.B., Shami A. (2020). Systematic ensemble model selection approach for educational data mining. Knowl. -Based Syst..

[15] Singh D., Ng B., Lai Y.-C., Lin Y.-D., Seah W.K. (2018). Modelling Software-Defined Networking: Software and Hardware Switches. J. Netw. Comput. Appl..

[16] Krongbaramee P., Somchit Y. Implementation of SDN Stateful Firewall on Data Plane Using Open VSwitch. Proceedings of the 2018 15th International Joint Conference on Computer Science and Software Engineering (JCSSE).

[17] Lockwood J.W., McKeown N., Watson G., Gibb G., Hartke P., Naous J., Raghuraman R., Luo J. NetFPGA–An Open Platform for Gigabit-Rate Network Switching and Routing. Proceedings of the 2007 IEEE International Conference on Microelectronic Systems Education (MSE’07).

[18] Lu G., Guo C., Li Y., Zhou Z., Yuan T., Wu H., Xiong Y., Gao R., Zhang Y. ServerSwitch: A Programmable and High Performance Platform for Data Center Networks. Proceedings of the 8th USENIX Symposium on Networked Systems Design and Implementation (NSDI 11).

[19] Anwer M.B., Motiwala M., Tariq M.B., Feamster N. Switchblade: A Platform for Rapid Deployment of Network Protocols on Programmable Hardware. Proceedings of the ACM SIGCOMM 2010 Conference.

[20] Medved J., Varga R., Tkacik A., Gray K. Opendaylight: Towards a Model-Driven Sdn Controller Architecture. Proceedings of the IEEE International Symposium on a World of Wireless, Mobile and Multimedia Networks.

[21] Itoh T., Sakai M., Okada M. (2011). Floodlight. Google Patents.

[22] Gude N., Koponen T., Pettit J., Pfaff B., Casado M., McKeown N., Shenker S. (2008). NOX: Towards an Operating System for Networks. ACM SIGCOMM Comput. Commun. Rev..

[23] Tootoonchian A., Ganjali Y. Hyperflow: A Distributed Control Plane for Openflow. Proceedings of the 2010 Internet Network Management Conference on Research on Enterprise Networking.

[24] Prakash A., Priyadarshini R. An Intelligent Software Defined Network Controller for Preventing Distributed Denial of Service Attack. Proceedings of the 2018 Second International Conference on Inventive Communication and Computational Technologies (ICICCT).

[25] Srivastava R., Richhariya V. (2013). Survey of Current Network Intrusion Detection Techniques. J. Inf. Eng. Appl..

[26] Kumari K., Prasad A., Prasad K. (2016). Dielectric, Impedance/Modulus and Conductivity Studies on [Bi0. 5 (Na1-XKx) 0.5] 0.94 Ba0. 06TiO_3_, (0.16 ≤ *x* ≤ 0.20) Lead-Free Ceramics. Am. J. Mater. Sci.

[27] Wu H., Schwab S., Peckham R.L. (2008). Signature Based Network Intrusion Detection System and Method. Google Patents.

[28] Yulianto A., Sukarno P., Suwastika N.A. (2019). Improving Adaboost-Based Intrusion Detection System (IDS) Performance on CIC IDS 2017 Dataset. J. Phys. Conf. Ser..

[29] Liao H.-J., Lin C.-H.R., Lin Y.-C., Tung K.-Y. (2013). Intrusion Detection System: A Comprehensive Review. J. Netw. Comput. Appl..

[30] Vigna G., Kemmerer R.A. (1999). NetSTAT: A Network-Based Intrusion Detection System. J. Comput. Secur..

[31] Sekar R., Guang Y., Verma S., Shanbhag T. A High-Performance Network Intrusion Detection System. Proceedings of the 6th ACM Conference on Computer and Communications Security.

[32] Hoque M.S., Mukit M., Bikas M., Naser A. (2012). An Implementation of Intrusion Detection System Using Genetic Algorithm. arXiv.

[33] Gales G. (2003). Network Intrusion Detection System and Method. Google Patents.

[34] Brownlee J. (2016). Supervised and Unsupervised Machine Learning Algorithms. Mach. Learn. Mastery.

[35] Bonaccorso G. (2017). Machine Learning Algorithms.

[36] Zamani M., Movahedi M. (2013). Machine Learning Techniques for Intrusion Detection. arXiv.

[37] Vishwanathan S., Narasimha M.M. SSVM: A Simple SVM Algorithm. Proceedings of the Proceedings of the 2002 International Joint Conference on Neural Networks, IJCNN’02 (Cat. No.02CH37290).

[38] El Naqa I., Murphy M.J. (2015). What Is Machine Learning?.

[39] Tsai C.-F., Hsu Y.-F., Lin C.-Y., Lin W.-Y. (2009). Intrusion Detection by Machine Learning: A Review. Expert Syst. Appl..

[40] Liaw A., Wiener M. (2002). Classification and Regression by RandomForest. R News.

[41] Vapnik V.N., Vapnik V. (1998). Statistical Learning Theory.

[42] Yekkehkhany B., Safari A., Homayouni S., Hasanlou M. (2014). A Comparison Study of Different Kernel Functions for SVM-Based Classification of Multi-Temporal Polarimetry SAR Data. Int. Arch. Photogramm. Remote Sens. Spat. Inf. Sci..

[43] Bao J., Nie J., Liu C., Jiang B., Zhu F., He J. (2019). Improved Blind Spectrum Sensing by Covariance Matrix Cholesky Decomposition and RBF-SVM Decision Classification at Low SNRs. IEEE Access.

[44] Steinwart I., Christmann A. (2008). Support Vector Machines.

[45] Martínez-Ramón M., Christodoulou C. (2005). Support Vector Machines for Antenna Array Processing and Electromagnetics. Synth. Lect. Comput. Electromagn..

[46] Zwane S., Tarwireyi P., Adigun M. A Flow-Based IDS for SDN-Enabled Tactical Networks. Proceedings of the 2019 International Multidisciplinary Information Technology and Engineering Conference (IMITEC).

[47] Eid H.F., Darwish A., Hassanien A.E., Abraham A. Principle Components Analysis and Support Vector Machine Based Intrusion Detection System. Proceedings of the 2010 10th International Conference on Intelligent Systems Design and Applications.

[48] Zanero S., Savaresi S.M. Unsupervised Learning Techniques for an Intrusion Detection System. Proceedings of the 2004 ACM Symposium on Applied Computing.

[49] Cover T., Hart P. (1967). Nearest Neighbor Pattern Classification. IEEE Trans. Inf. Theory.

[50] Syarif I., Prugel-Bennett A., Wills G. (2012). Unsupervised Clustering Approach for Network Anomaly Detection. Proceedings of the International Conference on Networked Digital Technologies.

[51] Kohonen T. (1998). The Self-Organizing Map. Neurocomputing.

[52] Hulle V. (2012). Self-Organizing Maps. Handb. Nat. Comput..

[53] Hastie T., Tibshirani R., Friedman J. (2001). The Elements of Statistical Learning. Springer Series in Statistics.

[54] Kanungo T., Mount D.M., Netanyahu N.S., Piatko C.D., Silverman R., Wu A.Y. (2002). An Efficient K-Means Clustering Algorithm: Analysis and Implementation. IEEE Trans. Pattern Anal. Mach. Intell..

[55] Haweliya J., Nigam B. (2014). Network Intrusion Detection Using Semi Supervised Support Vector Machine. Int. J. Comput. Appl..

[56] Chen C., Gong Y., Tian Y. Semi-Supervised Learning Methods for Network Intrusion Detection. Proceedings of the 2008 IEEE International Conference on Systems, Man and Cybernetics.

[57] Lee D.-H. Pseudo-Label: The Simple and Efficient Semi-Supervised Learning Method for Deep Neural Networks. Proceedings of the Workshop on Challenges in Representation Learning.

[58] Wu H., Prasad S. (2017). Semi-Supervised Deep Learning Using Pseudo Labels for Hyperspectral Image Classification. IEEE Trans. Image Process..

[59] Chapelle O., Scholkopf B., Zien A. (2009). Semi-Supervised Learning. IEEE Trans. Neural Netw..

[60] Vamvoudakis K.G. (2017). Q-Learning for Continuous-Time Linear Systems: A Model-Free Infinite Horizon Optimal Control Approach. Syst. Control Lett..

[61] Arulkumaran K., Deisenroth M.P., Brundage M., Bharath A.A. (2017). Deep Reinforcement Learning: A Brief Survey. IEEE Signal Process. Mag..

[62] Li Y. (2017). Deep Reinforcement Learning: An Overview. arXiv.

[63] He Y., Liang C., Richard Y.F., Han Z. (2018). Trust-Based Social Networks with Computing, Caching and Communications: A Deep Reinforcement Learning Approach. IEEE Trans. Netw. Sci. Eng..

[64] D’Oro S., Galluccio L., Palazzo S., Schembra G. (2017). A Game Theoretic Approach for Distributed Resource Allocation and Orchestration of Softwarized Networks. IEEE J. Sel. Areas Commun..

[65] Narmanlioglu O., Zeydan E. Learning in SDN-Based Multi-Tenant Cellular Networks: A Game-Theoretic Perspective. Proceedings of the 2017 IFIP/IEEE Symposium on Integrated Network and Service Management (IM).

[66] Gao Y., Cheng L., Sang L., Yang D. Spectrum Sharing for LTE and WiFi Coexistence Using Decision Tree and Game Theory. Proceedings of the 2016 IEEE Wireless Communications and Networking Conference.

[67] Shi H.-Y., Wang W.-L., Kwok N.-M., Chen S.-Y. (2012). Game Theory for Wireless Sensor Networks: A Survey. Sensors.

[68] Agrawal A., Jaiswal D. (2012). When Machine Learning Meets Ai and Game Theory. Comput. Sci..

[69] Ranadheera S., Maghsudi S., Hossain E. (2017). Mobile Edge Computation Offloading Using Game Theory and Reinforcement Learning. arXiv.

[70] Salama M.A., Eid H.F., Ramadan R.A., Darwish A., Hassanien A.E. (2011). Hybrid Intelligent Intrusion Detection Scheme.

[71] Fiore U., Palmieri F., Castiglione A., Santis D. (2013). Network Anomaly Detection with the Restricted Boltzmann Machine. Neurocomputing.

[72] Thaseen S., Kumar C.A. An Analysis of Supervised Tree Based Classifiers for Intrusion Detection System. Proceedings of the 2013 International Conference on Pattern Recognition, Informatics and MOBILE Engineering.

[73] Goodfellow I., Bengio Y., Courville A. (2016). Deep Learning.

[74] Bengio Y., Goodfellow I., Courville A. (2017). Deep Learning.

[75] Bharati A., Singh R., Vatsa M., Bowyer K.W. (2016). Detecting Facial Retouching Using Supervised Deep Learning. IEEE Trans. Inf. Secur..

[76] Ren Z., Yan J., Ni B., Liu B., Yang X., Zha H. Unsup75ervised Deep Learning for Optical Flow Estimation. Proceedings of the Thirty-First AAAI Conference on Artificial Intelligence.

[77] Taylor A., Leblanc S., Japkowicz N. Anomaly Detection in Automobile Control Network Data with Long Short-Term Memory Networks. Proceedings of the 2016 IEEE International Conference on Data Science and Advanced Analytics (DSAA).

[78] Kang M.-J., Kang J.-W. (2016). Intrusion Detection System Using Deep Neural Network for In-Vehicle Network Security. PLoS ONE.

[79] Kim J., Shin Y., Choi E. (2019). An Intrusion Detection Model Based on a Convolutional Neural Network. J. Multimed. Inf. Syst..

[80] Kasongo S.M., Sun Y. (2019). A Deep Learning Method with Filter Based Feature Engineering for Wireless Intrusion Detection System. IEEE Access.

[81] Kim J., Kim J., Huong T., Kim H. Long Short Term Memory Recurrent Neural Network Classifier for Intrusion Detection. Proceedings of the International Conference on Platform Technology and Service (PlatCon).

[82] Yin C., Zhu Y., Fei J., He X. (2017). A Deep Learning Approach for Intrusion Detection Using Recurrent Neural Networks. IEEE Access.

[83] Tang T.A., Mhamdi L., McLernon D., Raza A., Ghogho M. Deep Recurrent Neural Network for Intrusion Detection in Sdn-Based Networks. Proceedings of the 4th IEEE Conference on Network Softwarization and Workshops (NetSoft).

[84] Nasr M., Bahramali A., Houmansadr A. Deepcorr: Strong Flow Correlation Attacks on Tor Using Deep Learning. Proceedings of the 2018 ACM SIGSAC Conference on Computer and Communications Security.

[85] Zhang Y., Chen X., Jin L., Wang X., Guo D. (2019). Network Intrusion Detection: Based on Deep Hierarchical Network and Original Flow Data. IEEE Access.

[86] Yu Y., Long J., Cai Z. (2017). Network Intrusion Detection through Stacking Dilated Convolutional Autoencoders. Secur. Commun. Netw..

[87] Alrawashdeh K., Purdy C. Toward an Online Anomaly Intrusion Detection System Based on Deep Learning. Proceedings of the 2016 15th IEEE International Conference on Machine Learning and Applications (ICMLA).

[88] Egerstedt M. (2015). From algorithms to architectures in cyber-physical networks. Cyber-Phys. Syst..

[89] Gao N., Gao L., Gao Q., Wang H. An Intrusion Detection Model Based on Deep Belief Networks. Proceedings of the 2017 VI International Conference on Network, Communication and Computing Huangshan.

[90] Otoum S., Kantarci B., Mouftah H.T. (2019). On the Feasibility of Deep Learning in Sensor Network Intrusion Detection. IEEE Netw. Lett..

[91] Inayat U., Zia M.F., Mahmood S., Khalid H.M., Benbouzid M. (2020). Learning-Based Methods for Cyber Attacks Detection in IoT Systems: A Survey on Methods, Analysis, and Future Prospects. Electronics.

[92] Thamilarasu G., Chawla S. (2019). Towards Deep-Learning-Driven Intrusion Detection for the Internet of Things. Sensors.

[93] He Y., Mendis G.J., Wei J. (2017). Real-Time Detection of False Data Injection Attacks in Smart Grid: A Deep Learning-Based Intelligent Mechanism. IEEE Trans. Smart Grid.

[94] Shone N., Ngoc T.N., Phai V.D., Shi Q. (2018). A Deep Learning Approach to Network Intrusion Detection. IEEE Trans. Emerg. Top. Comput. Intell..

[95] Musleh A.S., Khalid H.M., Muyeen S.M., Al-Durra A. (2019). A Prediction Algorithm to Enhance Grid Resilience Toward Cyber Attacks in WAMCS Applications. IEEE Syst. J..

[96] Zhang H., Yu X., Ren P., Luo C., Min G. (2019). Deep Adversarial Learning in Intrusion Detection: A Data Augmentation Enhanced Framework. arXiv.

[97] Zhou L., Ouyang X., Ying H., Han L., Cheng Y., Zhang T. Cyber-Attack Classification in Smart Grid via Deep Neural Network. Proceedings of the 2nd International Conference on Computer Science and Application Engineering.

[98] Feng F., Liu X., Yong B., Zhou R., Zhou Q. (2019). Anomaly Detection in Ad-Hoc Networks Based on Deep Learning Model: A Plug and Play Device. Ad Hoc Netw..

[99] Zhang Y., Li P., Wang X. (2019). Intrusion Detection for IoT Based on Improved Genetic Algorithm and Deep Belief Network. IEEE Access.

[100] Jiang F., Fu Y., Gupta B.B., Liang Y., Rho S., Lou F., Meng F., Tian Z. (2018). Deep Learning Based Multi-Channel Intelligent Attack Detection for Data Security. IEEE Trans. Sustain. Comput..

[101] Zhang H., Wang Y., Chen H., Zhao Y., Zhang J. (2017). Exploring Machine-Learning-Based Control Plane Intrusion Detection Techniques in Software Defined Optical Networks. Opt. Fiber Technol..

[102] Aldwairi T., Perera D., Novotny M.A. (2018). An Evaluation of the Performance of Restricted Boltzmann Machines as a Model for Anomaly Network Intrusion Detection. Comput. Netw..

[103] Alom M.Z., Taha T.M., Yakopcic C., Westberg S., Sidike P., Nasrin M.S., Hasan M., Essen V., Awwal A.A., Asari V.K. (2019). A State-of-The-Art Survey on Deep Learning Theory and Architectures. Electronics.

[104] Yang J., Deng J., Li S., Hao Y. (2017). Improved Traffic Detection with Support Vector Machine Based on Restricted Boltzmann Machine. Soft Comput..

[105] Zhao G., Zhang C., Zheng L. Intrusion Detection Using Deep Belief Network and Probabilistic Neural Network. Proceedings of the 2017 IEEE International Conference on Computational Science and Engineering (CSE) and IEEE International Conference on Embedded and Ubiquitous Computing (EUC) Guangzhou.

[106] Khan F.A., Gumaei A., Derhab A., Hussain A. (2019). A Novel Two-Stage Deep Learning Model for Efficient Network Intrusion Detection. IEEE Access.

[107] Papamartzivanos D., Mármol F.G., Kambourakis G. (2019). Introducing Deep Learning Self-Adaptive Misuse Network Intrusion Detection Systems. IEEE Access.

[108] Marotta A., Carrozza G., Avallone S., Manetti V. An OpenFlow-Based Architecture for IaaS Security. Proceedings of the ATACCS ‘13: International Conference on Application and Theory of Automation in Command and Control Systems.

[109] Yasrebi P., Monfared S., Bannazadeh H., Leon-Garcia A. Security Function Virtualization in Software Defined Infrastructure. Proceedings of the 2015 IFIP/IEEE International Symposium on Integrated Network Management (IM).

[110] Carvalho L.F., Abrão T., Leonardo, Lemes M. (2018). An Ecosystem for Anomaly Detection and Mitigation in Software-Defined Networking. Expert Syst. Appl..

[111] Leland W.E., Willinger W., Taqqu M.S., Wilson D.V. (1995). On the Self-Similar Nature of Ethernet Traffic. ACM SIGCOMM Comput. Commun. Rev..

[112] Tang T.A., Mhamdi L., McLernon D., Raza A., Ghogho M. Deep Learning Approach for Network Intrusion Detection in Software Defined Networking. Proceedings of the 2016 International Conference on Wireless Networks and Mobile Communications (WINCOM).

[113] Latah M., Toker L. (2018). Towards an Efficient Anomaly-Based Intrusion Detection for Software-Defined Networks. IET Netw..

[114] Prasath M.K., Perumal B. (2019). A Meta-Heuristic Bayesian Network Classification for Intrusion Detection. Int. J. Netw. Manag..

[115] Kannadiga P., Zulkernine M. DIDMA: A Distributed Intrusion Detection System Using Mobile Agents. Proceedings of the Sixth International Conference on Software Engineering, Artificial Intelligence, Networking and Parallel/Distributed Computing and First ACIS International Workshop on Self-Assembling Wireless Network.

[116] Wang B., Sun Y., Yuan C., Xu X. LESLA: A Smart Solution for SDN-Enabled MMTC E-Health Monitoring System. Proceedings of the 8th ACM MobiHoc 2018 Workshop on Pervasive Wireless Healthcare Workshop.

[117] Ashraf S., Shawon M.H., Khalid H.M., Muyeen S.M. (2021). Denial-of-Service Attack on IEC 61850-Based Substation Automation System: A Crucial Cyber Threat towards Smart Substation Pathways. Sensors.

[118] Ashraf J., Latif S. Handling Intrusion and DDoS Attacks in Software Defined Networks Using Machine Learning Techniques. Proceedings of the 2014 National Software Engineering Conference.

[119] Kokila R., Thamarai S.S., Govindarajan K. DDoS Detection and Analysis in SDN-Based Environment Using Support Vector Machine Classifier. In Proceeding of the 2014 Sixth International Conference on Advanced Computing (ICoAC).

[120] Wang P., Chao K.-M., Lin H.-C., Lin W.-H., Lo C.-C. An Efficient Flow Control Approach for SDN-Based Network Threat Detection and Migration Using Support Vector Machine. Proceedings of the 2016 IEEE 13th International Conference on e-Business Engineering (ICEBE).

[121] Shiravi A., Shiravi H., Tavallaee M., Ghorbani A.A. (2012). Toward Developing a Systematic Approach to Generate Benchmark Datasets for Intrusion Detection. Comput. Secur..

[122] Gangadhar S., Sterbenz J.P. Machine Learning Aided Traffic Tolerance to Improve Resilience for Software Defined Networks. Proceedings of the 2017 9th International Workshop on Resilient Networks Design and Modeling (RNDM).

[123] Neupane R.L., Neely T., Chettri N., Vassell M., Zhang Y., Calyam P., Durairajan R. Dolus: Cyber Defense Using Pretense against DDoS Attacks in Cloud Platforms. Proceedings of the 19th International Conference on Distributed Computing and Networking.

[124] Nanda S., Zafari F., DeCusatis C., Wedaa E., Yang B. Predicting Network Attack Patterns in SDN Using Machine Learning Approach. Proceedings of the 2016 IEEE Conference on Network Function Virtualization and Software Defined Networks (NFV-SDN).

[125] Song C., Park Y., Golani K., Kim Y., Bhatt K., Goswami K. Machine-Learning Based Threat-Aware System in Software Defined Networks. Proceedings of the 2017 26th International Conference on Computer Communication and Networks (ICCCN).

[126] Alshamrani A., Chowdhary A., Pisharody S., Lu D., Huang D. A Defense System for Defeating DDoS Attacks in SDN Based Networks. Proceedings of the 15th ACM International Symposium on Mobility Management and Wireless Access.

[127] Smith R.J., Zincir-Heywood A.N., Heywood M.I., Jacobs J.T. Initiating a Moving Target Network Defense with a Real-Time Neuro-Evolutionary Detector. Proceedings of the 2016 on Genetic and Evolutionary Computation Conference Companion.

[128] Narayanadoss A.R., Truong-Huu T., Mohan P.M., Gurusamy M. Crossfire Attack Detection Using Deep Learning in Software Defined ITS Networks. Proceeding of the 2019 IEEE 89th Vehicular Technology Conference (VTC2019-Spring).

[129] Anderson S., Wickboldt J.A., Granville L.Z., Schaeffer-Filho A. ATLANTIC: A Framework for Anomaly Traffic Detection, Classification, and Mitigation in SDN. Proceedings of the NOMS 2016—2016 IEEE/IFIP Network Operations and Management Symposium.

[130] Kreutz D., Ramos F.M., Verissimo P. Towards Secure and Dependable Software-Defined Networks. Proceedings of the HotSDN ‘13: Second ACM SIGCOMM Workshop on Hot Topics in Software Defined Networking.

[131] Akhunzada A., Gani A., Anuar N.B., Abdelaziz A., Khan M.K., Hayat A., Khan S.U. (2016). Secure and Dependable Software Defined Networks. J. Netw. Comput. Appl..

[132] Wang Y., Cai W., Wei P. (2016). A Deep Learning Approach for Detecting Malicious JavaScript Code. Secur. Commun. Netw..

[133] Sommer R., Paxson V. Outside the Closed World: On Using Machine Learning for Network Intrusion Detection. Proceedings of the IEEE Symposium on Security and Privacy.

[134] Petroulakis N.E., Spanoudakis G., Askoxylakis I.G. (2016). Patterns for the Design of Secure and Dependable Software Defined Networks. Comput. Netw..

[135] Prete L.R., Shinoda A.A., Schweitzer C.M., Santos Simulation in an SDN Network Scenario Using the POX Controller. Proceedings of the 2014 IEEE Colombian Conference on Communications and Computing (COLCOM).

[136] Wehrle K., Günes M., Gross J. (2010). Modeling and Tools for Network Simulation.

[137] Fontes R.R., Afzal S., Brito S.H., Santos M.A., Rothenberg C.E. Mininet-WiFi: Emulating Software-Defined Wireless Networks. Proceedings of the 2015 11th International Conference on Network and Service Management (CNSM).

[138] Hande Y., Muddana A. (2021). A Survey on Intrusion Detection System for Software Defined Networks (SDN).

[139] Elsayed M.S., Le-Khac N.-A., Dev S., Jurcut A.D. Ddosnet: A Deep-Learning Model for Detecting Network Attacks. Proceedings of the 2020 IEEE 21st International Symposium on “A World of Wireless, Mobile and Multimedia Networks” (WoWMoM).

[140] Zargar S.T., Joshi J., Tipper D. (2013). A Survey of Defense Mechanisms against Distributed Denial of Service (DDoS) Flooding Attacks. IEEE Commun. Surv. Tutor..

[141] Kolias C., Kambourakis G., Stavrou A., Voas J. (2017). DDoS in the IoT: Mirai and Other Botnets. Computer.

[142] Su A.-J., Choffnes D.R., Kuzmanovic A., Bustamante F.E. (2006). Drafting behind Akamai (Travelocity-Based Detouring). ACM SIGCOMM Comput. Commun. Rev..

[143] Khalid H.M., Muyeen S.M., Peng J.C.-H. (2020). Cyber-Attacks in a Looped Energy-Water Nexus: An Inoculated Sub-Observer-Based Approach. IEEE Syst. J..

[144] Giotis K., Argyropoulos C., Androulidakis G., Kalogeras D., Maglaris V. (2014). Combining OpenFlow and SFlow for an Effective and Scalable Anomaly Detection and Mitigation Mechanism on SDN Environments. Comput. Netw..

[145] Lim S., Ha J., Kim H., Kim Y., Yang S. A SDN-Oriented DDoS Blocking Scheme for Botnet-Based Attacks. Proceedings of the 2014 Sixth International Conference on Ubiquitous and Future Networks (ICUFN).

[146] Liu Z., He Y., Wang W., Zhang B. (2019). DDoS Attack Detection Scheme Based on Entropy and PSO-BP Neural Network in SDN. China Commun..

[147] Winter P., Hermann E., Zeilinger M. Inductive Intrusion Detection in Flow-Based Network Data Using One-Class Support Vector Machines. Proceedings of the 2011 4th IFIP International Conference on New Technologies, Mobility and Security.

[148] Trung V., Huong T.T., Tuyen V., Duc D.M., Thanh N.H., Marshall A. A Multi-Criteria-Based DDoS-Attack Prevention Solution Using Software Defined Networking. Proceedings of the 2015 International Conference on Advanced Technologies for Communications (ATC).

[149] Jadidi Z., Muthukkumarasamy V., Sithirasenan E., Sheikhan M. Flow-Based Anomaly Detection Using Neural Network Optimized with GSA Algorithm. Proceedings of the 33rd International Conference on Distributed Computing System.

[150] Niyaz Q., Sun W., Javaid A.Y. (2016). A Deep Learning Based DDoS Detection System in Software-Defined Networking (SDN). arXiv.

[151] Dawoud A., Shahristani S., Raun C. A Deep Learning Framework to Enhance Software Defined Networks Security. Proceedings of the 32nd IEEE International Conference on Advanced Information Networking and Applications Workshops: IEEE WAINA 2018.

[152] Khalid H.M., Peng J.C.-H. (2016). A Bayesian Algorithm to Enhance the Resilience of WAMS Applications Against Cyber Attacks. IEEE Trans. Smart Grid.

[153] Team M. (2012). Mininet an Instant Virtual Network on Your Laptop (or Other PC). https://ic.unicamp.br.

[154] Shu J., Zhou L., Zhang W., Du X., Guizani M. (2020). Collaborative Intrusion Detection for VANETs: A Deep Learning-Based Distributed SDN Approach. IEEE Trans. Intell. Transp. Syst..

[155] Mauro D., Galatro G., Liotta A. (2020). Experimental Review of Neural-Based Approaches for Network Intrusion Management. IEEE Trans. Netw. Serv. Manag..

[156] Garg S., Kaur K., Kumar N., Rodrigues J.J. (2019). Hybrid Deep-Learning-Based Anomaly Detection Scheme for Suspicious Flow Detection in SDN: A Social Multimedia Perspective. IEEE Trans. Multimed..

[157] Creech G., Hu J. Generation of a New IDS Test Dataset: Time to Retire the KDD Collection. Proceedings of the 2013 IEEE Wireless Communications and Networking Conference (WCNC).

[158] Moustafa N., Slay J. (2016). The Evaluation of Network Anomaly Detection Systems: Statistical Analysis of the UNSW-NB15 Data Set and the Comparison with the KDD99 Data Set. Inf. Secur. J. A Glob. Perspect..

[159] Lee J., Pak J., Lee M. Network Intrusion Detection System Using Feature Extraction Based on Deep Sparse Autoencoder. Proceedings of the 2020 International Conference on Information and Communication Technology Convergence (ICTC).

[160] Mauro D., Galatro G., Fortino G., Liotta A. (2021). Supervised Feature Selection Techniques in Network Intrusion Detection: A Critical Review. Eng. Appl. Artif. Intell..

[161] Coates A., Ng A., Lee H. An Analysis of Single-Layer Networks in Unsupervised Feature Learning. Proceedings of the Fourteenth International Conference on Artificial Intelligence and Statistics.

[162] Lu Y., Cohen I., Zhou X.S., Tian Q. Feature Selection Using Principal Feature Analysis. Proceedings of the Proceedings of the 15th ACM International Conference on Multimedia.

[163] Eid H.F., Salama M.A., Hassanien A.E., Kim T. (2011). Bi-Layer Behavioral-Based Feature Selection Approach for Network Intrusion Classification.

[164] Kloft M., Brefeld U., Düessel P., Gehl C., Laskov P. (2008). Automatic Feature Selection for Anomaly Detection. https://ml.cs.uni-kl.de/publications/aisec08-kloft.pdf.

[165] Gogoi P., Bhuyan M.H., Bhattacharyya D., Kalita J.K. (2012). Packet and Flow Based Network Intrusion Dataset.

[166] Axelsson S. (1998). Research in Intrusion-Detection Systems: A Survey.

[167] Alom M.Z., Bontupalli V., Taha T.M. Intrusion Detection Using Deep Belief Networks. Proceedings of the 2015 National Aerospace and Electronics Conference (NAECON).

[168] Yan Q., Richard Y.F., Gong Q., Li J. (2015). Software-Defined Networking (SDN) and Distributed Denial of Service (DDoS) Attacks in Cloud Computing Environments: A Survey, Some Research Issues, and Challenges. IEEE Commun. Surv. Tutor..

[169] Aburomman A.A., Reaz M.B. Survey of Learning Methods in Intrusion Detection Systems. Proceedings of the 2016 International Conference on Advances in Electrical, Electronic and Systems Engineering (ICAEES).

